# The effects of psychosocial stress on intergroup resource allocation

**DOI:** 10.1038/s41598-019-54954-w

**Published:** 2019-12-09

**Authors:** Adam Schweda, Nadira Sophie Faber, Molly J. Crockett, Tobias Kalenscher

**Affiliations:** 1 Comparative Psychology, Heinrich-Heine-University Düsseldorf, Düsseldorf, Germany; 2 College of Life and Environmental Sciences, University of Exeter, Exeter, United Kingdom; 3 Oxford Uehiro Centre for Practical Ethics, University of Oxford, Oxford, United Kingdom; 4 Department of Psychology, Yale University, New Haven, Connecticut USA

**Keywords:** Social behaviour, Stress and resilience, Human behaviour

## Abstract

Stress changes our social behavior. Traditionally, stress has been associated with “fight-or-flight” – the tendency to attack an aggressor, or escape the stressor. But stress may also promote the opposite pattern, i.e., “tend-and-befriend” – increased prosociality toward others. It is currently unclear which situational or physiological factors promote one or the other. Here, we hypothesized that stress stimulates both tendencies, but that fight-or-flight is primarily directed against a potentially hostile outgroup, moderated by rapid-acting catecholamines, while tend-and-befriend is mainly shown towards a supportive ingroup, regulated by cortisol. To test this hypothesis, we measured stress-related neurohormonal modulators and sex hormones in male and female participants who were exposed to a psychosocial stressor, and subsequently played an intergroup social dilemma game in which they could reveal prosocial motives towards an ingroup (ingroup-love) and hostility towards an outgroup (outgroup-hate). We found no significant effects of stress on social preferences, but stress-related heart-rate increases predicted outgroup-hostile behavior. Furthermore, when controlling for testosterone, cortisol was associated with increased ingroup-love. Other-regarding behavior was overall higher in male than female participants. Our mixed results are of interest to scholars of the effects of stress on prosocial and aggressive behavior, but call for refinement in future replications.

## Introduction

Stress is known to alter social behavior. The canonical social response to stress is fight-or-flight^1^. Fight-or-flight responses prepare an organism for homeostasis for antagonistic situations^2^, thus increasing the individual’s propensity to aggress and flee. The fight-or-flight response to stress is a well-documented phenomenon that has been widely observed in humans and non-human animals^3^. For example, in humans, stress has been shown to reduce empathy^4^ and financial generosity^5^ to foster domestic and general violence^6,7^ and to promote aggressive criminal behavior^8^. Aggressive fight-or-flight responses go along with arousal, activation of the sympathetic nervous system and mobilization of energy resources, and they are linked to rapid-acting catecholaminergic, mainly noradrenergic (NA) components of the stress response^1,9–12^.

However, recent theoretical and empirical evidence suggests that stress can also induce prosocial behavior^13^. For example, von Dawans and colleagues^14^ found that psychosocial stress increased males’ trust in others and their sharing of monetary resources. This was interpreted as support for the “tend-and-befriend”^13^ hypothesis. The tendency to “tend-and-befriend” is a proclaimed coping strategy that involves investing into social networks after stress, thus offering costly help to a delimited group of people in order to seek and offer mutual protection during anticipated or experienced threats^13–17^. The tend-and-befriend hypothesis, initially only postulated as being female-specific^16^, has received empirical support in recent years. For example, stress has been shown to increase acceptance of even unfair offers in the ultimatum game amongst women^18^. A tend-and-befriend response has been found in males, too. For instance, stress has been shown, across sexes, to increase donation rates among participants with pro-environmental attitudes^19^, to increase trust and sharing behavior in male participants^14^, and generosity in males^20,21^. Consistent with the tend-and-befriend hypothesis, stressed individuals report more social closeness^22^, socio-evaluative stress has been shown to enhance emotional empathy^23^, and empathy- and prosociality-related brain areas are activated after a hybrid stress task^24^. Furthermore, consistent with the assumption of tending-and-befriending a close social network under stress, induction of psychosocial stress leads to increased generosity towards socially close recipients of help, for instance relatives and friends, but not towards socially distant others, such as strangers^20^.

There is suggestive evidence that prosocial tend-and-befriend responses to stress are linked to relatively slow-acting cortisol (CORT), a component of the physiological stress response that is distinct from fast-acting catecholamines. For example, exogenous manipulation of CORT activity has been shown to foster financial altruism towards socially close others^21^, and stress-related CORT-levels covary with greater trust^25^ and social affiliation^22^. The idea that the separate components of the physiological stress response, NA and CORT, have dissociable effects on social behavior has been supported by the recent observation that CORT-related financial altruism could be counteracted by additional administration of yohimbine (an alpha2-adrenoceptor antagonist that boosts NA release^21^). This discovery is in line with the finding that noradrenergic activity correlates negatively with overall financial generosity^20^ and even implicit intergroup bias^26–28^.

Thus, existing evidence suggests stress can promote aggressive (fight-or-flight) as well as prosocial tendencies (tend-and-befriend), and these two tendencies are tentatively related to distinct components of the physiological stress response. However, it is currently unclear when and why stressed individuals show tend-and-befriend or fight-or-flight behavior. Here, we propose that stress does not provoke one or the other response, but boosts both tendencies at the same time by supporting prosocial behavior towards socially close others (tend-and-befriend), who, unlike strangers, can potentially provide comfort and support in stressful times^20,21^. Simultaneously, stress could foster aggression against socially distant outgroup members who are more likely to present a threat than ingroup members (fight-or-flight). Because of recent evidence for a role of CORT in promoting generosity towards others^20,21,24,25^, and the classic association of fight-or-flight tendencies with sympathetic activation, we further hypothesize that tend-and-befriend and fight-or-flight tendencies are modulated by the dissociable actions of the stress-neuromarkers CORT and NA, with CORT promoting prosociality towards ingroup members, and NA fostering aggressive behavior against outgroup members.

Stress induces a complex, non-linear and time-dependent suite of neurohormonal changes. CORT and NA exhibit different response profiles, with NA peaking shortly after stress onset, and CORT roughly 20–30 minutes later^29^. CORT effects on neural activity can further be categorized into faster-acting non-genomic CORT action and slower, but longer-lasting (up to several days in animals) genomic CORT effects^30^. Here, participants play the IPD-MD within a 10-minute time window after offset of the gTSST, at the time at which we expect CORT and NA-action to overlap^31^.

Moreover, since male participants are known to respond to stress differently than females^32,33^ and often reveal different, gender-dependent social preferences^34,35^ we additionally considered gender in our main analyses, as well as the sex hormones testosterone, estradiol and progesterone^36–39^, and a range of other state and trait variables.

To test these hypotheses, we induced psychosocial stress in 100 male and 102 female participants (total n = 202), using the group version of the Trier Social Stress Test (gTSST^40^). After performing the gTSST or control procedures, participants played an adapted version of the intergroup prisoner’s dilemma maximizing differences game (IPD-MD^41^). In this game, three participants were assigned to one group, and they were told they would play against another group that participated on the previous day. To manipulate group affiliation, participants were instructed that the members of their own group held similar political views (ingroup), and that the members of the other group held radically opposing political views (outgroup^42^). At the beginning of the game, each participant received an initial economic endowment, which they could distribute across three pools (keep-pool, within-group pool and between-group pool). Contributions to the keep-pool would be kept by the participants; 50% of the total sum of contributions to the within-group pool would be paid out to each in-group member, including the participant; contributions to the between-group pool had the same effect to the ingroup, but each outgroup member would lose the amount each ingroup member received (see Table 1 for payoff matrix and example). Hence, contributions to the keep-pool can be interpreted as the motivation to maximize own profit (own-utility maximizing), and contributions to the within-group pool can be interpreted as costly motivation to maximize ingroup profit (called “ingroup love” in the relevant literature^41–47^). Finally, contributions to the between-group pool can be interpreted as motivation to maximize ingroup profit and, at the same time, harm the outgroup (so called “outgroup-hate”). Note that we opted against including a “pure spite” condition that would allow participants to harm the outgroup without giving benefits to the ingroup, as costly spite in the absence of ingroup favoritism occurs very rarely, if ever, in the laboratory or the field^48,49^.

**Table 1:** Payoff matrix of the IPD-MD for each pool separately. Outcomes are displayed for each player after player 1 from the ingroup invests 5€ (example).

		Player 1	Player 2	Player 3
Keep pool	Ingroup	+5€*	0€	0€
*Player invests 5€	Outgroup	0€	0€	0€
Within-Group Pool	Ingroup	+2.50€*	+2.50€	+2.50€
*Player invests 5€	Outgroup	0€	0€	0€
Between-Group Pool	Ingroup	+2.50€*	+2.50€	+2.50€
*Player invests 5€	Outgroup	−2.50€	−2.50€	−2.50€

We predicted that stressed participants would contribute more money to the between-group pool than non-stressed participants, reflecting the predicted combination of ingroup-love and outgroup-hate, and that contributions to this pool would be correlated with measures of the sympathetic stress response (salivary marker of NA and heart-rate). Furthermore, we expected that the motivation to contribute to either the within-group or the between-group pool over keep-pool investments would be correlated with the amplitude of the salivary CORT response, reflecting the predicted CORT effects on prosociality. Although our stress induction was successful, as indicated by physiological and subjective stress markers, our results did not confirm these hypotheses, at least not unambiguously. While we found evidence for an association between heart-rate increase (indicating sympathetic stress response) and outgroup-hate, the stress manipulation did not significantly affect overall preferences, and there was no correlation between salivary CORT measures and pool investments. However, CORT predicted within-group pool contributions when testosterone was controlled for. Overall, these results suggest a complex relation between stress and intergroup rivalry.

## Results

### Main results

#### Trait measures and group-differences in hormones

To rule out systematic stress-unrelated differences between participants of the stress and control groups, we collected a range of individual trait measures. None of the trait measures differed significantly between groups, with the exception of chronotype and a marginally significant group difference in chronic stress. Moderation analyses with these two factors as potential moderators revealed no significantly influencing role on any of the outcome variables. In addition, there were no significant differences in any of the sex hormone measures (testosterone, progesterone and estradiol) between participants of the stress and control group (cf. SOM for details and analyses).

#### Manipulation check of stress induction

**Figure 1:**
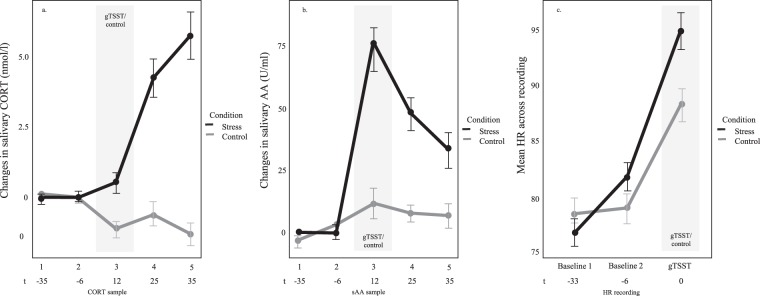
Physiological stress markers. (**a**) Baseline-corrected cortisol (CORT) increased in the stress group, but not the control group (*p* < 0.001). Its peak was reached 20–30 minutes after gTSST onset. (**b**) Stress increased baseline-corrected salivary α-amylase (sAA; *p* < 0.001). SAA increased with stressor-onset. (**c**) Heart-rate increase was more pronounced in stressed than non-stressed participants (*p* < 0.001). All error bars indicate ± 1 SEM.

Compared to controls, participants in the gTSST group had significantly elevated CORT and salivary α-amylase (a measure of central noradrenergic activity^50^) levels (see Fig. 1). In addition, the subjective stress measures also revealed higher levels of psychological stress, such as negative affect, feelings of shame, and insecurity (cf. SOM).

#### IPD-MD: Main analyses

**Figure 2:**
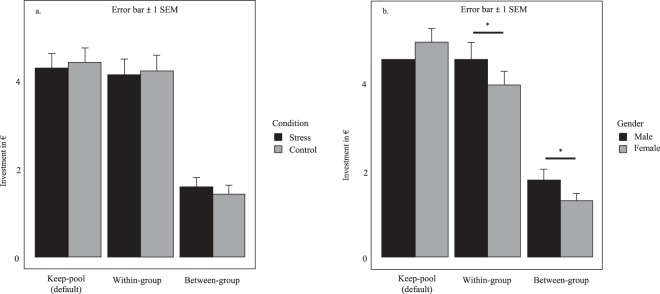
Contributions to the keep-, within-group and between-group pool in the IPD-MD. (**a**) Although participants made more keep- and within-group than between-group investments (more egoism and ingroup-love than outgroup-hate), psychosocial stress did not alter investment patterns. (**b**) Main effect of gender on pool investments. Male participants invested more into the within-group and the between-group pools than females, irrespective of whether they underwent the gTSST procedure or not, suggesting more other-regarding behavior in male than female participants (*p* = 0.03).

To assess our main hypotheses, we computed mixed ANOVAs to test for effects of condition (stress vs. control) and gender on investments into the within-group and between-group pools. A priori power analyses for the effect of stress on allocation patterns yield 95% power for at least medium-sized effects (see methods). Consistent with earlier findings^41,45,51^, subjects invested more into the within-group pool (*M* = 4.17, *SD* = 3.55) than the between-group pool (*M* = 1.51, *SD* = 2.16, main effect of pool: *F*(1, 197) = 59.54, *p* < 0.001, *η*_p_^2^ = 0.232, see Fig. 2). Male participants contributed more to both pools than female participants, i.e., female participants kept more money for themselves (keep-pool; males: *M* = 3.1, *SD* = 3.58, females: *M* = 2.58, *SD* = 2.82, main effect of gender: *F*(1, 197) = 4.79, *p* = 0.030, *η*_p_^2^ = 0.024, see Fig. 2). However, there were no significant interaction effects between stress, gender and pool (gender x condition: *F*(1, 197) = 0.02, *p* = 0.879, *η*_p_^2^ < 0.001; gender x pool: *F*(1, 197) = 0.04, *p* = 0.837, *η*_p_^2^ < 0.001; gender x pool x condition: *F*(1, 197) = 0.12, *p* = 0.724, *η*_p_^2^ = 0.001). Contrary to our predictions, stress did neither significantly increase within-group, nor between-group pool investments (main effect of condition: *F*(1, 197) = 0.04, *p* = 0.844, *η*_p_^2^ < 0.001, 95% CI [0, 0.019]; interaction effect condition x pool: *F*(1, 197) = 0.13, *p* = 0. 0.720, *η*_p_^2^ = 0.001, 95% CI [0, 0.025]). Please note that the latter effect sizes’ 95% confidence interval upper bound can still be considered a small effect.

**Figure 3:**
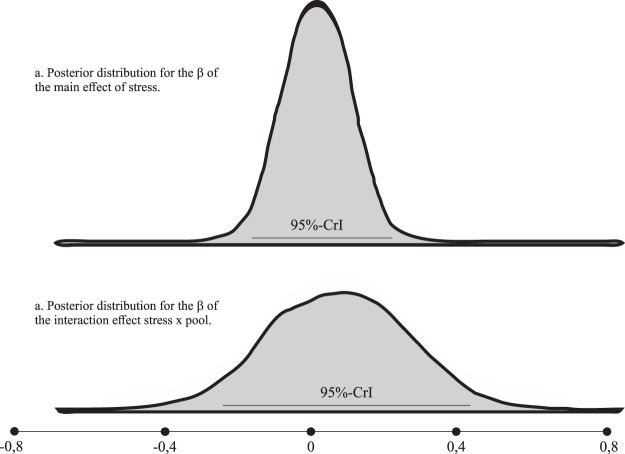
Posterior distributions of the effects of stress on IPD-MD contributions. Horizontal lines mark 95% credibility intervals. (**a**) posterior probability distribution for the standardized regression coefficient of the effect of stress; a marked deviation from zero would indicate that stress influences both, within-group and between-group pool investments, in some, but the same direction. However, it is centered around the mean of 0.02 and the 95% credibility intervals are bounded at −0.16 and 0.20, indicating that – given a 95% criterion – the standardized difference is unlikely to be larger than 0.20 (effect size at the boundary of the heavier tail). (**b**) posterior probabilities of the interaction term pool x condition. Heavy deviations from zero would indicate that stress affects within- and between-group pool investments differentially, for example by only increasing outgroup-hate. Although the posterior mean is close to zero (0.08) and the left tail’s 95%-CrI bounds at −0.28, the posterior distribution is right-tailed with an interval boundary at 0.43. This very likely results from a slight, but, in frequentistic terms, statistically insignificant increase of between-pool investments in the stress vs. the control group.

To receive a realistic distribution of plausible stress-related effects and, hence, further elucidate our null finding, we additionally computed Bayesian credibility intervals, which have been shown to produce high coverage of true parameters^52^. To this end, the main effect of condition and its interaction with pool were estimated in a full Bayesian mixed linear model (see SOM for details). The results indicate comparably narrow credibility intervals around zero for stress-related effects, except for the slight increase of investments into the between-group pool (pool: β = −0.82, 95%-CrI [−1.00, −0.64]; condition: β = 0.02, 95%-CrI [−0.16, 0.20]; pool x condition: β = 0.08, 95%-CrI [−0.28, 0.43]). The posterior distributions for condition, pool and their interaction are displayed in Fig. 3. Equivalence tests (“region of practical equivalence”, ROPE^53^) based on the stress-related posteriors’ 95%-highest density intervals (HDI) accept the null hypothesis if defined as HDIs congruent with an interval of ~20% (for the main effect of stress) or ~45% (for the interaction pool x stress) of the grand standard deviation around zero^53^.

To further corroborate these null results, we applied Bayesian hypothesis testing to obtain a quantitative estimate of the evidence for the null hypothesis, using a model comparison approach (see SOM for details). For the following calculations, we set up a mixed linear model with pool and condition as fixed effects and a varying intercept per subject (see SOM for details). Taking into account the reverse of the Bayes factor in favor of the alternative hypothesis - the BF01 - we find evidence for our null hypothesis (only condition: BF01 = 8.523, “moderate” evidence^54^; condition and the interaction term condition x pool: BF01 = 51.476, “very strong” evidence^54^), and this result remains stable for a wider array of prior definitions (see SOM for prior robustness checks). Hence, given the centeredness of the posterior distributions of the stress-related effect-estimates around zero, as well as the Bayes Factors for the null hypothesis, the most reasonable conclusion is that stress does either have no or only a small effect on IPD-MD investments.

#### Stress markers, sex hormones and investment patterns in the IPD-MD

Next, we asked if investment decisions in the IPD-MD were moderated by changes in the levels of stress markers CORT, α-amylase and heart-rate, independent of a main effect of stress. To this end, we regressed stress marker estimates (α-amylase and CORT, and heart-rate) on the contributions to the pools. As estimates, we considered the area under the curve with respect to increase (AUCi^55^) for CORT and α-amylase, and the increase in heart-rate from the average of the two baseline recordings to the gTSST/control procedure. A mixed linear model with pool, stress markers CORT, α-amylase and HR, as well as their interactions with pool and condition as fixed effects was calculated. Intercepts varied per subject. Our results revealed that changes in heart-rate, but not CORT or α-amylase, modulated decision behavior. We found a significant interaction effect between pool and heart-rate increase (β = 0.196, *t(*298) = 3.141, *p* = 0.002). Simple regressions on the between- and within-group pool separately indicate an association between heart-rate increase and decreasing ingroup-friendly, as well as increasing outgroup-hostile investments (within-group pool: β = −0.238, *t*(149) = −2.223, *p* = 0.028, uncorrected; between-group pool: β = 0.153, *t*(149) = 2.41, *p* = 0.017, uncorrected). There were no main or interaction effects of α-amylase or CORT on pool investments (all p > 0.20.). Additional robust Bayesian parameter estimations revealed identical results (cf. SOM).

This finding is partly consistent with one of our main hypotheses that sympathetic activity, with heart-rate as a proxy, should be related to an increased tendency to cause outgroup harm. However, our second proxy of sympathetic activity, α-amylase estimates, was not significantly correlated with outgroup harm, thus limiting our ability to make a definite decision on our hypothesis.

None of the sex hormones testosterone, progesterone & estradiol, nor their interactions explained variance in IPD-MD contributions (cf. SOM). Inspired by the dual-hormone hypothesis^56,57^ that predicts an interaction effect of CORT and testosterone on behavior, we investigated if any of the sex hormones, particularly testosterone, moderated (hidden) effects of CORT on IPD-MD decisions. For interpretability, we constructed three different mixed linear models in an exploratory analysis in which we regressed pool (within-/between-group), the respective sex hormone, the area under the curve of CORT, and their interaction terms on the investments. In order to condition on participants’ gender and control for different effects of the sex hormones for males and females, we additionally entered gender as a factor. We let the intercept vary for each participant. Only the model including testosterone yielded significant findings: when testosterone was considered in the model, CORT increase (AUCi), as well as testosterone levels predicted pool investments. CORT levels were associated with an increase of allocations into the within-group pool, and testosterone boosted both within- and between-group pool investments (interaction pool x CORT β = −0.149, *t*(329) = −2.444, p = 0.015; testosterone β = 0.207, *t*(329) = 2.039, *p* = 0.042). CORT itself reached marginal significance (β = 0.115, *t*(329) = 1.876, *p* = 0.062). This suggests that CORT and testosterone levels explained the variance in within- and between-group investments that was not accounted for by each hormone alone. There was no significant interaction between testosterone and CORT on pool investments (β = −0.018, *t*(329) = 0.291, *p* = 0.711), testosterone and pool (β = −0.044, *t*(329) = −0.808, *p* = 0.420), nor any of the higher order interactions (all p > 0.17). Also, gender has not reached significance in this model (gender β = 0.107, *t*(329) = 1.104, *p* = 0.270). Robust Bayesian models show similar results (cf. SOM). Thus, in summary, we found that heart-rate increase predicted a shift from within- to between-group pool investments, and that CORT and testosterone levels explained the variance in within- and between-group investments if considered in a model that conditions on both hormones. This finding is partly consistent with our hypothesis predicting a double-dissociation of sympathetic activity and CORT on ingroup-love (within-group investments) and outgroup-hate (between-group investments). However, it has to be interpreted with caution because of the complexity of the results and the inconsistency in redundant stress marker effects, e.g., the lack of correlation of α-amylase with pool investments.

## Exploratory Analyses

### Sex-hormonal underpinnings of gender differences in IPD-MD investments

We ran additional exploratory analyses to further elucidate the gender differences in pool investments described above, suggesting that male participants invested more into both the within- and between-group pool than females (see SOM for details). We asked if these gender differences in behavior can be explained by diverging sex hormone profiles or trait measures that we collected. However, these analyses revealed that the gender differences in pool investments were unrelated to differences in the sex hormone compositions and trait measures, except for the emerging value of testosterone as a predictor when cortisol is considered (see above). Since this result needs to be considered with caution, and sex hormones are not directly related to pool investments, our data suggest that males’ higher contributions to both the within- and between-group pool likely reflected factors not considered in this study such as, for example, physiology-independent gender differences or same-sex group composition.

### IPD-MD investments and chronic stress

It is possible that chronic, but not acute stress (as induced by the gTSST procedure), altered investment behavior in the IPD-MD. Indeed, we found that an increase in chronic stress – as measured by the Trier Inventory of Chronic Stress^58^ (TICS; see SOM) - was associated with an overall decrease in ingroup-love and outgroup-hate, and thus, in other-regarding preferences (TICS β = −0.089, *t*(394) = −2.024, *p* = 0.043). This suggests that chronic stress, as opposed to acute psychosocial stress, is related to an overall disengagement from other-regarding investments. See SOM for analyses on other trait variables.

## Discussion

We measured the effects of psychosocial stress on social decision-making in an intergroup rivalry setting. We predicted that stress would increase ingroup-love, and, at the same time, promote outgroup-hate. We further expected that the prosocial effects of stress towards the ingroup would be related to the CORT-component of the neuroendocrine stress response, while aggressive tendencies of outgroup harm would be associated with the sympathetic part of the stress response, mainly NA action. We further considered the potentially moderating role of a range of other endocrine, trait and state variables, including gender, and sex hormones. Although our results are mixed, as discussed below, we found no straightforward support for our hypotheses.

Regarding our main hypothesis, there was no significant effect of psychosocial stress on ingroup-love, outgroup-hate, or selfish choice. A priori power analyses indicated sufficient power to capture small to medium-sized effects with our IPD-MD design. The Bayesian credibility interval of the main effect of stress on investments into the within- and between-pool is considerably narrow; if the heavier tailed 95%-bound of the stress-related posterior distributions of the β-estimates is considered an upper limit of a standardized measure of difference (β = 0.20), it only yields small plausible effects^59^, if any. The posterior distribution of the interaction term is tailed towards higher credibility. Here, plausible effect sizes based on the 95% bound of the longer tail (β = 0.43) of the posterior still range in the medium category^59^. Further calculations of Bayes factors show reasonable evidence in favor of our null hypotheses. This, as well as the narrow frequentist confidence intervals around the effect sizes, point to no – or a non-detectable - effect of our stress manipulation on IPD-MD decisions.

Of course, this analysis still leaves room for doubt of a true null effect. We could accept the HDI-based null hypothesis using criteria of 20% (for the main effect of stress) or 45% (for the interaction between pool and stress) of the standard deviation around 0 in an equivalence test, but these criteria are still very liberal^53^. The variability in individual contributions to the pools was large, so that we cannot exclude the possibility with certainty that we simply failed to detect small or noisy stress effects on choice. However, our sample size is large, and given our initial power, the centeredness of the posterior distributions, and the Bayes Factor analyses the most likely interpretation of our results is that psychosocial stress had very small or non-existent effects on investment behavior in the IPD-MD game.

We further hypothesized that outgroup-hate was linked to catecholaminergic activity, so that catecholamine action, especially noradrenaline, would boost between-group investments. Our secondary analyses yielded at least partial evidence for this hypothesis. Heart-rate increase from baseline to stress, which is heavily influenced by catecholaminergic action^60,61^, predicted a decrease of investment into the within-group pool, and a slight, statistically significant increase into the between-group pool. This suggests that sympathetic activity correlates with decreased ingroup-love, consistent with previous findings^21^, as well as increased outgroup-hate. We note that heart-rate responses to stress were associated with reduced prosociality and enhanced harm infliction in the absence of a main effect of stress on social decision-making.

However, while sympathetic activity is associated with enhanced NA release, our other marker of NA activity, α-amylase, did not significantly correlate with pool investments in the IPD-MD game. Hence, the question remains why heart-rate, but not α-amylase levels, predicted changes in ingroup-love and outgroup-hate. The heart-rate response is a temporally well-resolved measure of sympathetic activity, including, but not restricted to, noradrenergic release, and is also to a degree influenced by the parasympathetic nervous system^60^. α-amylase is mainly secreted by the parotid glands, it is directly controlled by sympathetic input, and linked to plasma noradrenaline^50,62^. Correlations between sympathetic indicators (such as skin conductance level^63^ and ventricular ejection time^64^) and α-amylase are moderate^50^ and often noisy. Also in our study, heart-rate and α-amylase correlate significantly, but weakly (*r* = 0.19, *p* = 0.018). Thus, both measures show complex relationships with sympathetic activation, and each have their own caveats^65,66^ because they measure different subprocesses of arousal. This might be one reason why other studies found behavioral measures to be correlated with one marker, but not the other^20^. Thus, the relationship between the sympathetic stress response and a shift away from ingroup-love to outgroup-hate is possibly real, but the specific mechanisms are complex and need to be illuminated in future studies.

We further hypothesized that the CORT response to the stress manipulation would be correlated with ingroup-love^21,25^. Neither stress nor CORT were directly associated with ingroup-love, but when controlling for the variance explained by testosterone, CORT indeed positively predicted within-group pool investments. Also, if testosterone levels were conditioned on CORT, testosterone predicted an increase in overall within- and between-group pool contributions. Note that, because the default option was to keep the investment (contributions to the keep-pool; see methods), increased investments into within- or between-group pools reflect a dominance of other-regarding over selfish motives. Hence, this finding suggests that, when considering the variance explained by either hormone, CORT and testosterone indeed predict ingroup-love and other-regarding behavior.

At first glance, this result seems consistent with the dual-hormone hypothesis^42,53^. This hypothesis postulates that testosterone needs low CORT to predict aggression, while CORT would boost empathy, but only at high testosterone levels^54^. However, despite the fact that, here, CORT levels predicted IPD-MD decisions only when controlling for testosterone (and vice versa), we found no statistical interaction between CORT and testosterone, nor any three-way interaction with pool; our results therefore cannot be readily interpreted as a CORT-testosterone moderation effect on social choice. Thus, our results are, once more, exploratory, complex and call for further investigation.

Not unexpectedly, male participants revealed more outgroup-hate, but also more ingroup-love (higher within- and between-group than keep investments) than female participants. Hence, male participants showed more other-regarding behavior while females were more selfish. This finding is consistent with much of the literature on gender differences in cooperative and competitive behavior, showing that men are often more competitive than women, but can also form cooperative alliances to reach a common goal and protect resources and social status^34,67–71^. The ultimate cause of such behaviors is often discussed in terms of the evolutionary importance of forming strong male bonds to enhance chances of success in intergroup conflicts^70^. Especially testosterone has been linked to rapidly and adaptively increasing the drive for competition in social settings^32^. The fact that we found a simultaneous increase in generosity and hostility in males might conform with possible bidirectionalities caused by testosterone. For example, exogenous testosterone administration has been found to both increase^61^ and decrease^72^ generosity in the ultimatum game. However, the evidence that testosterone mediated the gender effects on other-regarding behavior in the present study was weak: none of our sampled sex hormones were directly correlated with the male participants’ increased ingroup-love and outgroup-hate, except when testosterone and CORT were considered in one exploratory model, and none of our trait measures, such as psychopathy or social value orientation, explained the gender-dependent variance in IPD-MD choices. Hence, the proximal mechanisms underlying the gender differences in other-regarding behavior in the present study remain elusive and might be caused by other factors, such as non-physiological gender differences, that were not considered in this study. For example, a recent meta-analysis on the effects of same- versus mixed-sex group compositions on social behavior found that female participants are slightly less cooperative than men (d = 0.16) in same-sex settings^73^, suggesting that non-biological, environmental factors matter in shaping prosocial attitudes. Moreover, the interpretation of the small size of the gender effect (partial eta² = 0.024) requires caution, too.

Interestingly, exploratory analyses reveal an association between subjective chronic stress and an overall disengagement from other-regarding investments. Chronic stress has been frequently discussed as a trigger for social idleness, or even aggression; for example, rodents exposed to chronic physical stressors show a shutdown in social motivation, as well as antagonistic behavior against conspecifics^3,74^, and in humans, depressive periods that correlate with chronic stress might be accompanied by social isolation^75^, and sometimes even with sudden aggressive outbursts^76^. Of course, the correlative nature of our finding prompts caution, but if chronic stress could be identified as causal to decline of social engagement, this would shed light on the causes of some of the core symptoms of chronic stress disorders, and it would even have significant implications for our understanding of societal cohesion. Future studies should investigate the relationship between chronic stress in social and intergroup contexts e.g. by using modern approaches to causal inference in longitudinal data. Also, note that caution is required here because of our result’s exploratory nature, and the inherent potential for false-positive conclusions. Yet, our result is consistent with evidence from the animal literature suggesting increased social apathy, or even agression, with chronic stress^3^.

The most robust result of the present study is the null effect of psychosocial stress on pool investment in the IPD-MD game. The absence of any acute stress effect on social decision making is puzzling, given the vast number of studies, including experiments from our own lab, that found such effects on social choice^14,17,18,20–22,24,77,78^. It is unlikely that the null-effect of stress on social choices was due to ineffective or insufficient stress induction because all physiological and psychological stress markers indicate the contrary, i.e., successful stress induction in our participants. There are several other reasons why our manipulation might have failed to work. For instance, it is plausible that our implementation of the IPD-MD was not sufficiently sensitive to the social constructs it was supposed to measure, despite recent claims to the contrary with other implementations^42,79^. For instance, our task might have prompted deliberate, strategic thinking, but relied to a much lesser degree on social sentiments, such as ingroup-love and outgroup-hate^80,81^. That is, our participants’ predominant motive might have been payoff maximization, achieved by risk assessments and reciprocity expectations, and an attempt to balance these to make an optimal decision. Hence, social feelings like ingroup-love and outgroup-hate might have been overshadowed by these strategies, or they might have been outright irrelevant for task performance. Additional analyses (cf. SOM) partially support this possibility, suggesting that the IPD-MD might indeed be a predominantly strategic decision game, and less as an instrument to capture affectively tinted ingroup-love and outgroup-hate.

In addition, it is also possible that our ingroup-outgroup manipulation was not salient enough to produce true intergroup rivalry. We used political voter preferences to induce social group affiliation. Although our political framing induced relatively strong outgroup harm (between-subject pool investments) as compared to other studies (15.1% in our study vs. 6% in the original work by Halevy and colleagues), it was, overall, still low (a recent replication^82^ also reached 18.33% on average). And only half of our participants allocated money into the between-group pool at all. Consistent with Weisel and Böhm^42^, we conclude that political rivalries in Germany may not be strong enough to produce a robust outgroup harm response. The expression of any covert tendencies for outgroup harm might, additionally, be shackled by prevailing social norms prohibiting interpersonal aggression. Furthermore, it is uncertain whether similar political preferences generate the group cohesion necessary to produce ingroup affiliation, or even ingroup-love. Future studies could replicate the present experiment with a more emotionally salient intergroup manipulation aimed at overcoming harm aversion, such as the recruitment of members of minorities^83^ or rivalling ethnic tribes^84^.

Also, the temporal dynamics of the stress response need to be considered in future research. Here, participants performed the IPD-MD directly after stressor offset where CORT and NA-action are supposed to act in concert^31^. In order to obtain further evidence for our postulated dissociation between CORT for tend-and-befriend and NA for fight-or-flight, it would be interesting to administer the IPD-MD in different time windows after stress. If our hypothesis was true, then fight-or-flight tendencies should preferably occur during and shortly after the stressor, when catecholaminergic action peaks, while tend-and-befriend behavior should be predominantly found in the aftermath of stress during (genomic or non-genomic) CORT action. Also, future research should target the proximate neural mechanisms of “tend-and-befriend” and “fight-or-flight” in social decision making. Recent neuroimaging literature suggests that processes involving cooperation and intergroup cognition reliably recruit frontolimbic brain networks^85^. At the same time, acute stress was found to cause a massive reorganization of functional network dynamics in the brain, including the prefrontal cortex, the amygdala, as well as subcortical and orbitofrontal valuation-related areas^31,86,87^, suggesting that stress-related changes in those frontolimbic networks might underlie the promotion of tend-and-befriend or fight-or-flight tendencies. In addition, social decision making is known to be moderated by a range of other mental faculties, including reasoning, cognitive control, time-preference, memory and attention^88–94^ – faculties that are subserved by the same neural networks that are altered by stress. Hence, it is an open question if and how these mental functions and their underlying neural processes moderate or even mediate stress-effects on social behavior.

Overall, our study shows no direct effect of socio-evaluative stress on social decisions in the IPD-MD. However, heart-rate changes in response to stress were associated with a shift from ingroup-love to outgroup-hate. In addition, when considered in one model, CORT and testosterone action was associated with ingroup-love or both, ingroup-love and outgroup-hate - thus, other regarding behavior. In general, male participants revealed more other-regarding preferences than female participants, and this gender-effect was neither explained directly by sex hormones nor gender-dependent traits. Our study contributes to the literature on stress and decision making by highlighting the boundaries of stress effects on social choice; we conclude that our version of the IPD-MD failed to capture stress-related effects on social sentiments, probably due to its strategic character. Future studies are needed to further elucidate putative stress effects on social behavior.

## Methods

### Participants

One hundred and three male (age: *M* = 24.85, *SD* = 4.77), and 105 female participants (age: *M* = 24.71, *SD* = 5.13) were recruited within the Düsseldorf (Germany) area. Three male and three female subjects were excluded, either for incidentally knowing other members in their group (three females and two males), or for misunderstanding the intergroup prisoners’ dilemma rules, as revealed by the comprehension questions (one male). Based on a priori power analyses (G*Power^95^), we opted for a sample size that yielded 95% power for small to medium effect sizes for our pool x condition interaction (n = 206, Cohen’s *f* = 0.15, repeated measures correlation = 0.3). In case of a between-group effect, our experiment could detect effect sizes of 0.205 with a power of 0.95 (n = 204, repeated measures correlation = 0.3). With a final sample size of n = 202, our experiment is comparably well-powered.

Before participation, participants were screened via telephone interview for a number of eligibility criteria. Participants required to hold at least moderate sympathy for one of Germany’s five political parties with seats in the German parliament, except the right-wing populist party (*Alternative für Deutschland*, AfD). We applied a number of further eligibility criteria and participation rules, as outlined in the SOM.

The study was approved by the ethical committee of the Heinrich Heine University Düsseldorf, and our methods were performed in accordance with the committee’s rules and guidelines.

### Material

#### Trait measures and cognitive reflection

To exclude potential confounds and ensure similarity between participants in stress- and decision-making related traits, we collected a number of trait measures before the laboratory experiment and before the stress induction using online survey tools. These trait measures are described and summarized in the SOM.

#### Stress induction

All experimental sessions took place between 14:00 and 17:00 h to control for diurnal variation in CORT levels. We tested all participants in groups of three. They were randomly assigned to a stress condition or a control condition. Socio-evaluative stress was induced using the Trier Social Stress Test for groups^40^. In the stress condition, participants were exposed to a fictional job interview (net speaking time three minutes per participants) and, subsequently, to a mental arithmetic task in presence of other participants and in front of a non-responsive evaluation panel of experimenters, while their performance was video-taped. In the control condition, participants prepared a short talk about their friends, and they also performed an arithmetic task, but the evaluation panel paid ostensibly no attention to the participants. Participants spoke simultaneously and they were not videotaped. Stress and control conditions were matched in terms of cognitive load, speaking time, participant engagement etc., but differed in the socio-evaluative component^20,40^.

#### Physiological and subjective stress measures

**Figure 4:**
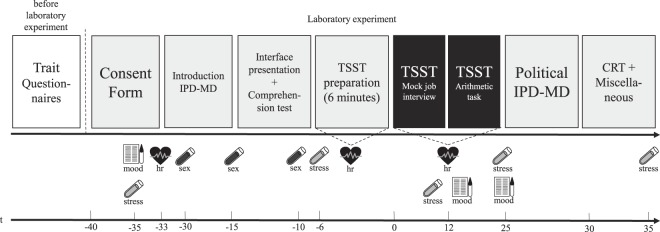
Illustration of experimental timeline. After giving the phone interview and completing the online questionnaires, participants were invited to the laboratory. All experimental sessions took place between 14:00 and 17:00. Participants gave informed consent and were familiarized with the different types of saliva sampling and the HR-monitor. Then, the first heart-rate baseline recording of 3 minutes started and the sex hormone and baseline CORT and α-amylase samples were collected together with the first PANAS measurement. The IPD-MD instructions were then given individually and comprehension was exhaustively tested, interspersed with the 2^nd^ and 3^rd^ sex-hormone samples. This was followed by heart-rate baseline monitoring for six minutes and gTSST/control procedure instructions, after which the gTSST/control procedure began. Participants completed the PANAS and gave further stress marker saliva samples during and directly after the gTSST/control procedure. Directly following the gTSST/control procedure, subjects played the IPD-MD. The IPD-MD lasted for no more than 10 minutes. The cognitive reflection task (CRT; cf. SOM), as well as a set of decision- and demographics-related questionnaires followed. 10 minutes after the TSST, we collected the last stress-marker saliva sample. The experiment concluded with a debriefing and individual, anonymous payouts.

We collected multiple saliva samples to determine stable baseline measures of the sex hormones progesterone, estradiol and testosterone, and to quantify the impact of our stress manipulation based on CORT and α-amylase. For the sex hormones, subjects filled ultra-pure polypropylene spit-in samples (SaliCaps, IBL International GmbH, Hamburg, Germany) with 1 mL of clear saliva. Three separate sex-hormone samples were collected in the first half of the experiment before subjection to the stress or control procedure (see Fig. 4). CORT/α-amylase samples (Salivette®, Sarstedt AG & Co. KG, Nuernbrecht, Germany) were collected throughout the entire experiment (see Fig. 4 for exact sample time points and SOM for a description of the timeline). Further details of saliva sampling and analysis procedures are provided in the SOM. As an additional measure of sympathetic activity, we recorded heart-rate using a commercially available HR-monitor (Polar A370, Polar Electro Oy, Kempele, Finland). Heart-rate was measured at several time points during the experiment (see Fig. 4 and SOM). Participants indicated positive and negative mood by completing a Positive and Negative Affect scale (PANAS^96^) before, during and after the gTSST/control procedure (see Fig. 4 and SOM), and, they also indicated current feelings of shame, insecurity, stress and confidence on visual analogue scales before, during and after the experimental procedures (VAS, 1–100).

### Intergroup prisoners’ dilemma - maximizing differences (IPD-MD)

We used political preferences to induce intergroup rivalry. Unlike other means of group manipulations^49^, group assignments based on political preferences have been shown to induce ingroup affiliation and outgroup harm while being realistic, feasible and credible^42^. The three participants in each testing session were instructed that they would form a group and play against another group of three other participants who performed the game one day before. They were told that group assignment was based according to the participants’ political voting preferences which were assessed before in the online questionnaires. The instructions explicitly stated that all members of the participants’ own group held similar political views (ingroup) and that the members of the other group were supporters of the political party “*Alternative für Deutschland*” (AfD; a German right-wing populist party). Outgroup decisions were not real and shammed by the experimenters. We used an adapted version of the IPD-MD to simulate intergroup behavior in the laboratory^41,42,79^. As mentioned above, in this game, two groups of three participants play against each other. Each participant receives the initial monetary endowment of 10 EUR, which they can freely distribute between three pools. Money contributed to the first pool (the “keep” pool), is kept by the player. For example, 5 EUR investment into the keep-pool would imply that the participant can keep those 5 EUR for herself. Fifty percent of the total sum of contributions to the second pool (the “within-group” pool) are paid out to each ingroup member, including the participant. For example, if a participant contributes 5 EUR to the within-group pool, each ingroup member, including the participant, would receive 2.50 EUR payback. Thus, contributions to the within-group pool are potentially costly to the participant because she only receives a back-payment of 50% of the invested sum if no one else contributes, but the overall sum of all payoffs to all group members is higher than individual contributions to the keep pool (the sum of the payoffs to all ingroup members in the above example is 7.50 EUR, see Table 1). Hence, the dominant group strategy^41^ would imply that every member contributes to the within-group pool since this would maximize the overall sum of payoffs to all players. For example, if all three ingroup members contributed their entire endowment of 10 EUR each to the within-group pool, the total sum of contributions would amount to 30 EUR, thus each ingroup member would receive a back-payment of 15 EUR.

Contributions to the third pool (the “between-group”-pool) have the same effect to the ingroup members as contributions to the within-group pool, but each outgroup member loses the amount each ingroup member receives. For example, if a participant invests 5 EUR to the between-group pool, each ingroup member, including the participant, would receive 2.50 EUR, and each outgroup member would lose 2.50 EUR. Thus, contributions to the between-group pool represent the same social dilemma as contributions to the within-group pool, but additionally entail the possibility to harm the outgroup.

Contributions to the keep-pool can be interpreted as the motivation to maximize own profit (own-utility maximizing), contributions to the within-group pool can be interpreted as potentially costly motivation to maximize ingroup profit (ingroup-love, or ingroup trust) and contributions to the between-group pool can be interpreted as costly motivation to maximize ingroup profit and, at the same time, harm the outgroup (outgroup-hate).

To ensure participants’ full understanding of the game, we presented extensive instructions covering multiple exemplary scenarios before stress induction. Subjects’ comprehension was controlled using a set of questions. If subjects revealed difficulties in understanding the rules of the game or its financial implications for the ingroup and outgroup, the respective parts of the game were explained again by the experimenters. The experiment was incentive-compatible. Subjects received a fixed participation fee of 20 EUR plus the gains from the in-group investments in the IPD-MD, minus 2 EUR as result of simulated between-group pool investments by the fictional outgroup decisions. Task completion took less than 10 minutes.

### Data analysis

To analyze IPD-MD distribution patterns, we applied a 2 × 2 × 2 mixed-factorial analysis of variance (ANOVA) with pool as a repeated-measures (within-group vs. between group; note that investments into all three pools were not independent, we therefore considered investments into the keep-pool as the default option and compared investments between the within- and between-group pools only) as well as condition (stress vs control) and gender (male vs female) as between-subject factors. To complement this analysis, we ran additional Bayes-Factor analyses on a mixed linear model with pool and condition as fixed factors and subject as a random intercept (using the software JASP^97^). Furthermore, full Bayesian parameter estimation of the same model was used to estimate posterior parameter distributions and to yield information on credible parameter values (R-Package *brms*^98^). We used the R-package *afex*^99^ for mixed linear models and ANOVAs.

### Ethics approval and consent to participate

The study was approved by the ethics committee of the Medical Faculty of the Heinrich-Heine-University in Dusseldorf. All participants gave their informed consent.

### Statement of responsibility

AS designed the task, ran the data collection, analyzed the data and wrote the paper, TK designed the task, wrote the paper, provided consultation at all stages of the project and funded the project, NF and MC edited the paper and provided consultation at all stages of the project^100–102^.

## Supplementary information

Supplementary Material
