# Supplementary material for: The effects of psychosocial stress on intergroup resource allocation
Source: Sci Rep. 2019 Dec 9;9:18620. doi: 10.1038/s41598-019-54954-w (PMC6901446; doi:10.1038/s41598-019-54954-w)
Supplement: Supplementary file 1 — Supplementary Material [file 41598_2019_54954_MOESM1_ESM.docx]

**The effects of psychosocial stress on intergroup resource allocation**

Schweda A.*^1^, Faber N. S. ^2^, Crockett M. J. ^3^, Kalenscher T. ^1^

Supplementary Information

**Supplemental Methods**

*Eligibility criteria for participant recruitment and participation rules*

Psychology or economics students were excluded from participation. We further excluded from participation: heavy smokers (>5 cigarettes), heavy drinkers (>3 portions of alcohol a day), regular drug users, non-german speakers, as well as overweight people (BMI > 30), people diagnosed with dyscalculia or psychiatric, neurological, endocrinological, urological, gynecological, cardiovascular conditions. To ensure endocrinological homogeneity, we only included heterosexuals^1^. To further control for hormonal effects on stress or choice behavior^2,3^, and for ethical reasons, we excluded pregnant female participants, and included only female participants who reported no irregularities in their menstrual cycle and no use of hormonal contraceptives during six months before participation. Subjects were told to avoid sexual intercourse, consumption of alcohol or medication 24 hours before the laboratory experiment. They ought to refrain from caffeine and cigarette consumption four hours, and food consumption and physical exercise four hours before the laboratory experiment. Groups consisted of three same-sex members. To reduce interference by circadian hormone fluctuations, we started each 90-minute session at 2 pm.

*Trait measures*

To exclude potential confounds and ensure similarity between participants in stress- and decision-making related traits, we collected a number of trait measures before the laboratory experiment and before the stress induction using online survey tools. We measured the level of identification with subjects' preferred political party and the extent of disfavor against other political parties (items based on Ohr, Quandt & Markus, 2011^4^), impulsiveness (Barratt Impulsiveness Scale, BIS-15^5^), approach and avoidance (Behavioral Inhibition / Activation Scale, BIS/BAS^6^), social desirability (Social Desirability Scale, SDS-17^7^), trait anxiety (Trait Scale of the State-Trait Anxiety Inventory, STAI^8^), social value orientation (number of socially-oriented decisions in the Triple Dominance Measure, SVO^9^), psychopathy (Levenson Self-Report Psychopathy Scale^10^), empathy (Interpersonal Reactivity Index^11^ of the Saarbruecker Persoenlichkeitsfragebogen, SPF^12^), individual chronotype (reduced version of the Morningness-Eveningness Questionnaire, rMEQ^13^), chronic stress (Trier Inventory of Chronic Stress, TICS^14^) and risk preference.

To investigate whether decision patterns in the IPD-MD were caused by changes in cognitive deliberation capacities, the three-item cognitive reflection task (CRT^15^) was applied directly after IPD-MD performance, thus after stress/control induction. The CRT is a well-established paradigm designed to quantify the ability to suppress intuitive, incorrect responses to simple arithmetic problems in favor of deliberate reasoning. CRT-performance has been shown to be CORT-sensitive^16^.

*Saliva sampling and analysis procedures*

We collected multiple saliva samples to determine stable baseline measures of the sex hormones progesterone, estradiol and testosterone, and to quantify the impact of our stress manipulation based on CORT and α-amylase. For the sex hormones, subjects filled ultra-pure polypropylene spit-in samples (SaliCaps, IBL International GmbH, Hamburg, Germany) with 1 mL of clear saliva. Three separate sex-hormone samples were collected in the first half of the experiment before subjection to the stress or control procedure (see figure 4 in the main paper). Sanguineous aliquots were excluded from analysis. All samples were stored at -26°C until analysis (conducted at Dresden Lab Service GmbH, Germany). Progesterone and testosterone levels were extracted using liquid chromatography with coupled tandem mass spectrometry (LC-MS/MS). Estradiol levels were obtained using a commercial immunoassay (17-beta-Estradiol Saliva Luminescence Immunoassay, IBL international, Hamburg). Due to high pulsatility of the sex hormones, the mean of the three samples was computed for statistical analyses. To determine saliva CORT and α-amylase levels, we collected two saliva samples before, one during and one approximately 10 minutes after the gTSST or control procuedures. We used polypropylene cotton swab samples (Salivette®, Sarstedt AG & Co. KG, Nuernbrecht, Germany). Subjects opened the aliquots, placed the cotton swab into their mouths and chewed on it for one minute. CORT and α-amylase samples were also stored at -26°C until analysis. Both α-amylase and CORT concentrations were determined using a luminescent immunoassay (IBL International, Hamburg).

**Supplemental Results**

*Manipulation check of stress induction I: physiological markers*

Mixed ANOVAs with saliva sample as a repeated measures factor, and condition and gender as between-subject factors revealed that salivary CORT was significantly higher in the stress than the control group, and increased in the stress group during gTSST performance relative to baseline (condition x sample interaction: *F*(1.73, 310.58) = 38.02, *p* < .001, η_p_^2^ = .174; main effect of condition: *F*(1, 180) = 15.18, *p*  < .001, η_p_^2^ = .078; and sample: *F*(1.73, 310.58) = 20.84, *p* < .001, η_p_^2^ = .104). There were no significant main or interaction effects of gender (main effect of gender: *F*(1, 180) = 0.22, *p* = .638, η_p_^2^ = .001; condition x gender: ; sample x gender: *F*(1, 180) =1.93, *p* = .167, η_p_^2^ = .011 ; gender x condition x sample: *F*(1.73, 310.58) = 0.77,*p* = .445, η_p_^2^ = .004).

The same analysis with α-amylase as dependent variable revealed that the gTSST significantly increased α-amylase (interaction condition x sample: *F*(2.5, 411.83) = 14.20, *p* < .001, η_p_^2^ = .079; main effect of condition *F*(1, 165) = 4.94, *p* = .028, η_p_^2^ = .029) and sample (*F*(2.5, 411.83) = 27.68, *p* < .001, η_p_^2^ = .144). Again, gender effects on α-amylase levels did not reach significance (main effect of gender: (*F*(1, 165) = 2.86, *p* = .09, η_p_^2^ = .02; condition x gender: *F*(1, 165) = 3.08, *p* = .08, η_p_^2^ = .02; sample x gender: *F*(2.5, 411.83) = 2.12, *p* = .109, η_p_^2^ = .013 ; gender x condition x sample: *F*(2.5, 411.83) = 0.62, *p* = .573, η_p_^2^ = .004). Finally, we found a significant increase in heart rate in participants performing the gTSST relative to control participants (*F*(1.84, 330.79) = 17.74, *p* < .001, η_p_^2^ = .090), accompanied by main effects of the within-subjects factor heart rate recording number (baseline 1, preparation, TSST, *F*(1.84, 330.79) = 262.18, *p* < .001, η_p_^2^ = .593) and the between subject factor condition (*F*(1, 180) = 1.74, *p* = .189, η_p_^2^ = .010). Females had a steeper increase in heart rate than males (*F*(1.84, 330.79) = 4.02, *p* = .022, η_p_^2^ = .022), but did not differ in overall heart rate (*F*(1, 180) = 0.17, *p* = .684, η_p_^2^ = .001). We found no 3-way interaction between gender, condition and recording on heart rate (*F*(1.84, 330.79) = 2.31, *p* = .105, η_p_^2^ = .013).

For further illustration, we plotted the raw-HR in supplementary figure 1.


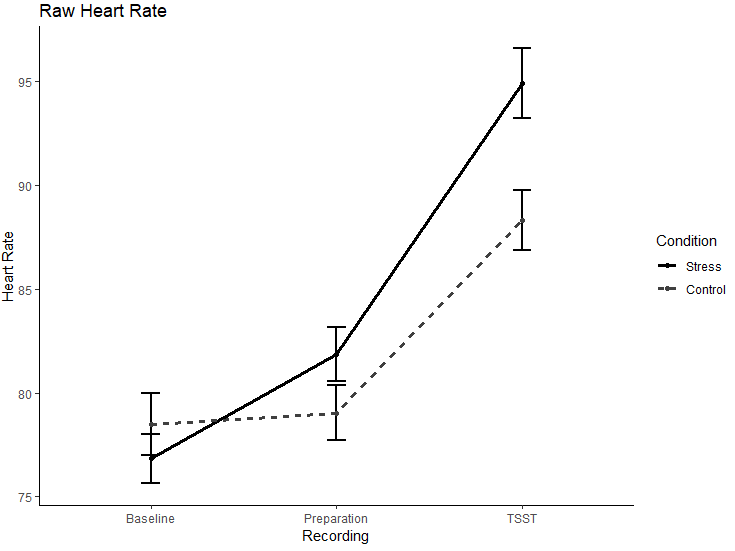


**Supplementary Figure 1: Raw-HR data.** In the main manuscript, we illustrated baseline-corrected values. For the sake of completeness, we present non-corrected HR here.

*Manipulation check of stress induction II: subjective measures*

We collected measures of subjective feelings of stress and mood before, during, and directly after the gTSST procedure. We ran a series of mixed ANOVAs to test for effects of condition, gender and measurement time points on the positive and the negative affect scale of the PANAS, feelings of shame, insecurity, stress, and confidence.

For the sake of brevity, we only shortly outline the VAS of stress-related feelings (see supplementary table 1). In the positive affect scale, we find an interaction effect between condition and time point (*F*(1.98, 389.83) = 27.43, *p* < .001, η_p_^2^ = .122). Simple effect analyses reveal increased positive affect ratings during the TSST (Stress: *M* = 23.202, *SD =* 4.123; Control: *M* = 20.294, *SD =* 3.709; *t(*320.85*)* = 5.533, *p* < .001) and after the TSST (Stress: *M* = 21.879, *SD =* 4.251; Control: *M* = 19.392, *SD =* 3.662; *t(*320.85*)* = 4.752, *p* < .001). Separate main effects of condition (*F*(1, 197) = 14.78, *p* < .001, η_p_^2^ = .070) and time point (*F*(1.98, 389.83) = 41.33, *p* < .001, η_p_^2^ = .173) also reached significance. Males and females did not differ in positive affect (main effect gender: *F*(1, 197) = 0.30, *p* = .584, η_p_^2^ = .002; gender x condition: *F*(1, 197) = 0.16, *p* = .686, η_p_^2^ = .001; gender x time point: *F*(1.98, 389.83) = 2.11, *p* = .124, η_p_^2^ = .011; gender x time point x condition: *F*(1.98, 389.83) = 0.45, *p* = .633, η_p_^2^ = .002).

The same pattern occurs in negative affect: We see an increase after the TSST stress condition (condition x time point: *F*(1.93, 380.48) = 24.41, *p* < .001, η_p_^2^ = .110), as well as separate effects of time point (*F*(1.93, 380.48) = 23.48, *p* < .001, η_p_^2^ = .107) and condition (*F*(1, 197) = 0.81, *p* = .368, η_p_^2^ = .004), but no involvement of gender (main effect gender: *F*(1, 197) = 2.15, *p* = .144, η_p_^2^ = .011; gender x time point: *F*(1.93, 380.48) = 2.05, *p* = .132, η_p_^2^ = .010; gender x condition: *F*(1, 197) = 0.81, *p* = .368, η_p_^2^ = .004; gender x condition x time point: *F*(1.93, 380.48) = 0.11, *p* = .891, η_p_^2^ = .001).

After stress, participants report marginally significant increases in feelings of shame (interaction condition x time point: *F*(1.99, 378.07) = 2.74, *p* = .066, η_p_^2^ = .014), as well as significant increases in insecurity (*F*(1.94, 368.98) = 13.79, *p* < .001, η_p_^2^ = .068), and feelings of stress as such (*F*(1.96, 371.98) = 18.14, *p* < .001, η_p_^2^ = .087). Also, a decrease in confidence during the TSST could be observed (interaction condition x time point: *F*(1.93, 367.44) = 2.97, *p* = .054, η_p_^2^ = .015) Female participants show overall higher levels of insecurity (main effect gender: *F*(1, 190) = 5.44, *p* = .021, η_p_^2^ = .028) and less confidence (*F*(1, 190) = 6.37, *p* = .012, η_p_^2^ = .032). All in all, physiological and subjective measures point towards a successful stress manipulation (see main text and supplementary table 1).

**Supplementary Table 1**

Comparisons of subjective stress measures between groups.

|  |  | Comparson between stress and control group | | | | |
| --- | --- | --- | --- | --- | --- | --- |
|  |  |  |  |  |  |  |
| Measurement | Number | Condition | Mean | SD | Statistic | p-Value |
|  |  |  |  |  |  |  |
| PANAS Positive Affect | I | Stress | 19.7 | 3.24 |  |  |
|  |  | Control | 19.8 | 3.17 | t = -0.237 | 0.814 |
|  | II | Stress | 23.2 | 4.12 |  |  |
|  |  | Control | 20.29 | 3.71 | t = 5.252 | < .001 |
|  | III | Stress | 21.88 | 4.25 |  |  |
|  |  | Control | 19.39 | 3.66 | t = 4.438 | < .001 |
| PANAS Negative Affect | I | Stress | 21.38 | 3.5 |  |  |
|  |  | Control | 22.08 | 3.72 | t = -1.382 | 0.169 |
|  | II | Stress | 24.48 | 4.26 |  |  |
|  |  | Control | 22.13 | 4.29 | t = 3.91 | < .001 |
|  | III | Stress | 23.25 | 4.54 |  |  |
|  |  | Control | 21.46 | 4.19 | t = 2.90 | 0.004 |
| VAS stressed | I | Stress | 25.44 | 21.74 |  |  |
|  |  | Control | 24.95 | 22.52 | t = 0.158 | 0.876 |
|  | II | Stress | 49.72 | 23.22 |  |  |
|  |  | Control | 32.72 | 23.94 | t = 4.99 | < .001 |
|  | III | Stress | 42.1 | 25.18 |  |  |
|  |  | Control | 28.26 | 23.23 | t = 4.05 | < .001 |
| VAS ashame | I | Stress | 9.73 | 14.88 |  |  |
|  |  | Control | 8 | 14.05 | t = 0.846 | 0.4 |
|  | II | Stress | 21.52 | 23.35 |  |  |
|  |  | Control | 15.26 | 22.09 | t = 1.92 | 0.056 |
|  | III | Stress | 21.38 | 24.61 |  |  |
|  |  | Control | 13.55 | 18.99 | t = 2.523 | 0.013 |
| VAS insecure | I | Stress | 19.6 | 18.65 |  |  |
|  |  | Control | 18.59 | 21.59 | t = 0.354 | 0.723 |
|  | II | Stress | 39.81 | 25.71 |  |  |
|  |  | Control | 23.94 | 22.57 | t = 4.567 | < .001 |
|  | III | Stress | 30.82 | 24.91 |  |  |
|  |  | Control | 20.57 | 20.14 | t = 3.2029 | 0.002 |
| VAS self-secure | I | Stress | 64.44 | 20.28 |  |  |
|  |  | Control | 64.01 | 20.63 | t = 0.151 | 0.88 |
|  | II | Stress | 54.4 | 21.63 |  |  |
|  |  | Control | 58.41 | 22.07 | t = -1.279 | 0.2026 |
|  | III | Stress | 56.4 | 22.23 |  |  |
|  |  | Control | 61.8 | 22.22 | t = -1.7221 | 0.09 |
|  |  |  |  |  |  |  |
|  |  |  |  |  |  |  |

*Manipulation check of induction of intergroup-bias and convergence with IPD-MD results I*

We included several measures to indirectly measure ingroup identification, and they indeed suggest that our group manipulation worked and that we could successfully create an intergroup bias. After the IPD-MD decision, we have collected a number of items that referred to participants’ expectations from the other players. Participants rated the following items on a 10-point Likert-scale:

1. In retrospect, how much did you expect your group (the other group) to invest for the money to be split up within your (their) group?
2. In retrospect, how much did you expect your group (the other group) to invest for the money to be deducted from the other (your) group?
3. Please rate: My motivation was to…
   1. … increase my own payoff.
   2. … increase my group’s payoff.
   3. … harm the other group.
4. Overall, how much hostility did you expect from the outgroup?

Furthermore, we measured group antipathy directed against ingroup and outgroup. Here, participants were asked how acceptable they would find a set of harmful actions towards others, such as interrupting them in a conversation, putting them at financial disadvantage, and hitting them. The table (Supplementary Table 2) below shows the results from either 2x2 ANOVAs with gender and stress if the respective items were not concerning in- and outgroup, or 2x2x2 ANOVAs with gender, stress condition and group if items concerned both, in- and outgroup.

**Supplementary Table 2**

*Additional control items referring to expectations of other players’ behavior during the IPD-MD. P-Values are not corrected for multiple comparisons.*

| Item |  | Marginal Descriptives | |  |  |  |  |
| --- | --- | --- | --- | --- | --- | --- | --- |
|  |  |  |  |  |  |  |  |
|  |  | M | SD | Effect | F | p |  |
| Expectation: investments -> within-group pool | Stress | 3.78 | 1.55 | Stress | 0.01 | 0.94 |  |
|  | Control | 3.78 | 1.61 | Gender | 6.44 | 0.01 |  |
|  | Male | 3.98 | 1.63 | Group | 9.82 | 0.002 |  |
|  | Female | 3.58 | 1.5 | Stress x Gender | 0.02 | 0.89 |  |
|  | Ingroup | 4.01 | 1.56 | Stress x Group | 0.34 | 0.56 |  |
|  | Outgroup | 3.55 | 1.57 | Gender x Group | 6.47 | 0.01 |  |
|  |  |  |  | Gender x Group x Stress | 0.61 | 0.44 |  |
|  |  |  |  |  |  |  |  |
| Expectation: investments -> between-group pool | Stress | 4.06 | 1.65 | Stress | 6.09 | 0.01 |  |
|  | Control | 4.5 | 1.75 | Gender | 2.56 | 0.11 |  |
|  | Male | 4.42 | 1.66 | Group | 17.84 | <.001 |  |
|  | Female | 4.14 | 1.76 | Stress x Gender | 0.01 | 0.93 |  |
|  | Ingroup | 3.96 | 1.61 | Stress x Group | 1.44 | 0.23 |  |
|  | Outgroup | 4.61 | 1.75 | Gender x Group | 1.18 | 0.28 |  |
|  |  |  |  | Gender x Group x Stress | 0 | 0.98 |  |
|  |  |  |  |  |  |  |  |
| Belief that outgroup acted in a hostile way | Stress | 4.2 | 1.67 | Stress | 0.17 | 0.68 |  |
|  | Control | 4.29 | 1.73 | Gender | 3.65 | 0.06 |  |
|  | Male | 4.48 | 1.71 | Condition x Gender | 2.57 | 0.11 |  |
|  | Female | 4.02 | 1.66 |  |  |  |  |
|  |  |  |  |  |  |  |  |
| My motivation was to… |  |  |  |  |  |  |  |
| ...increase my own payoffs | Stress | 5.01 | 1.57 | Stress | 1.14 | 0.29 |  |
|  | Control | 4.76 | 1.62 | Gender | 2.34 | 0.13 |  |
|  | Male | 5.06 | 1.54 | Condition x Gender | 0.12 | 0.73 |  |
|  | Female | 4.71 | 1.65 |  |  |  |  |
|  |  |  |  |  |  |  |  |
| ...increase payoffs of the group | Stress | 4.27 | 1.88 | Stress | 0.03 | 0.87 |  |
|  | Control | 4.31 | 1.82 | Gender | 0.18 | 0.67 |  |
|  | Male | 4.35 | 2.05 | Condition x Gender | 0 | 0.95 |  |
|  | Female | 4.24 | 1.63 |  |  |  |  |
|  |  |  |  |  |  |  |  |
| …harm the other group | Stress | 2.51 | 1.79 | Stress | 0.23 | 0.63 |  |
|  | Control | 2.38 | 1.8 | Gender | 0.14 | 0.71 |  |
|  | Male | 2.49 | 1.95 | Condition x Gender | 0.49 | 0.48 |  |
|  | Female | 2.4 | 1.63 |  |  |  |  |
|  |  |  |  |  |  |  |  |
|  |  |  |  |  |  |  |  |
| Group Antipathy | Stress | 13.56 | 6.77 | Stress | 0.03 | 0.87 |  |
|  | Control | 13.41 | 7.31 | Gender | 2.6 | 0.11 |  |
|  | Male | 14.15 | 7.54 | Group | 105.11 | <.001 |  |
|  | Female | 12.82 | 6.46 | Stress x Gender | 0.02 | 0.9 |  |
|  | Ingroup | 11.15 | 4.34 | Stress x Group | 0.03 | 0.86 |  |
|  | Outgroup | 15.82 | 8.35 | Gender x Group | 1.25 | 0.26 |  |
|  |  |  |  | Gender x Group x Stress | 0.01 | 0.93 |  |
|  |  |  |  |  |  |  |  |
|  |  |  |  |  |  |  |  |
|  |  |  |  |  |  |  |  |

Gender effects occur in the ingroups’ reciprocity expectation, at least when not correcting for multiple comparisons. Here, the interaction between gender and group is essential: Simple effects reveal that male participants expect more reciprocated within-group pool investments from the own investments than females (estimated marginal means: male M = 4.41, SE = 0.155, female M = 3.62, SE = 0.155, t(390) = 3.592, p < .001), but this does not pertain to expectations towards the outgroup (estimated marginal means: male M = 3.56, SE = 0.155, female M = 3.54, SE = 0.155, t(390) = 0.113, p = .910). Thus, in accordance with the gender effects on the within-pool investment in the main results, in contrast to females, male participants appear to believe that their co-participants would contribute more to the wellbeing of the group. However, we would like to stress that these results might arise due to alpha-error-inflation, and, at the very most, complement our main results, in which we found that male participants overall exhibited more other-regarding behavior.

As shown in the table (2), the effect of stress in the overall expectations of between-group pool investments reaches statistical significance, which could point towards an increased feeling of trust and security towards all other players. Yet again, this analysis concerns single items of a questionnaire, and p-values do not survive correction for multiple comparisons (applying the Bonferroni-method). Hence, we have only little, if any, evidence to assume that our stress manipulation affected the reciprocity expectation in the in- and the outgroup.

Overall, the most robust effect is, indeed, the ingroup-outgroup divergence in within-group and between-group pool investment expectancy, but also group antipathy. Participants expected their ingroup to invest more into the within-group pool than the other party. Furthermore, participants expected the outgroup to invest more into the between-group pool and act in a more hostile fashion than the ingroup. Lastly, people had more hostile feelings towards the outgroup, declaring more willingness to act harmfully on them. Altogether, these findings support our claim that the group manipulation worked, i.e., that we could create an intergroup bias in our experimental setting.

*Manipulation check of induction of intergroup-bias II: Did participants believe the cover story?*

First evidence that our participants believed the cover story is provided by the fact that group antipathy, as well as reciprocity expectation were consistent with the assumption of a real ingroup-outgroup effect: participants made above-zero investments into the within-group and between-group pool (within-group pool t(200) = 16.627, p < .001, between-group pool t(200) = 9.899, p < .001; see supplementary table 2 on reciprocity expectations).

More importantly, to further check whether belief in the cover story influenced decisions, participants rated the strength of the following convictions, using 10-point Likert scales:

1. During your decision, how much did you believe that your group’s players consisted of sympathisers of the same party you prefer?
2. During your decision, how much did you believe that the other group`s players consisted of AfD-voters who played a day before?

The histograms are shown in the figure (supplementary figure 2) below. The distribution of the responses to answer a. is slightly right skewed, and to answer b. reasonably uniform. Indeed, the sole question format is, in itself, highly susceptible for hindsight bias. In order to control if these measures had any effect on the pool investments, we conducted spearman correlations between responses on these two items and the pool investments. Only the belief that the other group was real (question a) correlated with investments into the within-group pool (rs = .156, p = .029). To further evaluate whether participants’ beliefs affected behavior in the IPD-MD, and perhaps even moderated the effect of stress, we constructed a full Bayesian robust mixed linear model with pool (within- vs. between-group), condition (stress vs. control), belief item a, belief item b, as well as various of their interactions terms with pool and condition (see supplementary table 3 below). Here, we found that the belief in the identity of the ingroup and outgroup critically impacted pool investments (see β-estimates of interaction outgroup belief x pool and ingroup belief x pool in table below). When breaking down these interactions into single regressions on each pool, belief in the ingroup’s identity actually decreased within-group pool investments (β = -.20, CrI: [-0.38 : -0.02]), but had no effect on between-group pool investments (although the β’s posterior was shifted into this direction, while still covering 0, β = 0.06, CrI = [-0.03 : 0.15]). Belief in the identity of the outgroup was, on the other side, positively associated with within-group pool investments (β = -.26, CrI: [-0.08 : -0.44]), but not between-group pool investments (β = -.05, CrI: [-0.13 : -0.03]), just as in the above mentioned correlation analysis.

Importantly, however, we found no evidence that the stress effect on IPD-MD decisions was moderated by belief. Indeed, parameter estimates for the main effect of stress and the interaction between stress and condition remain almost unaffected by entering variables concerning belief in ingroup and outgroup identity into the model. We therefore conclude that belief in the cover story had an effect on IPD-MD decisions to some extent, but it could not explain our (null) effect of stress on IPD-MD performance.


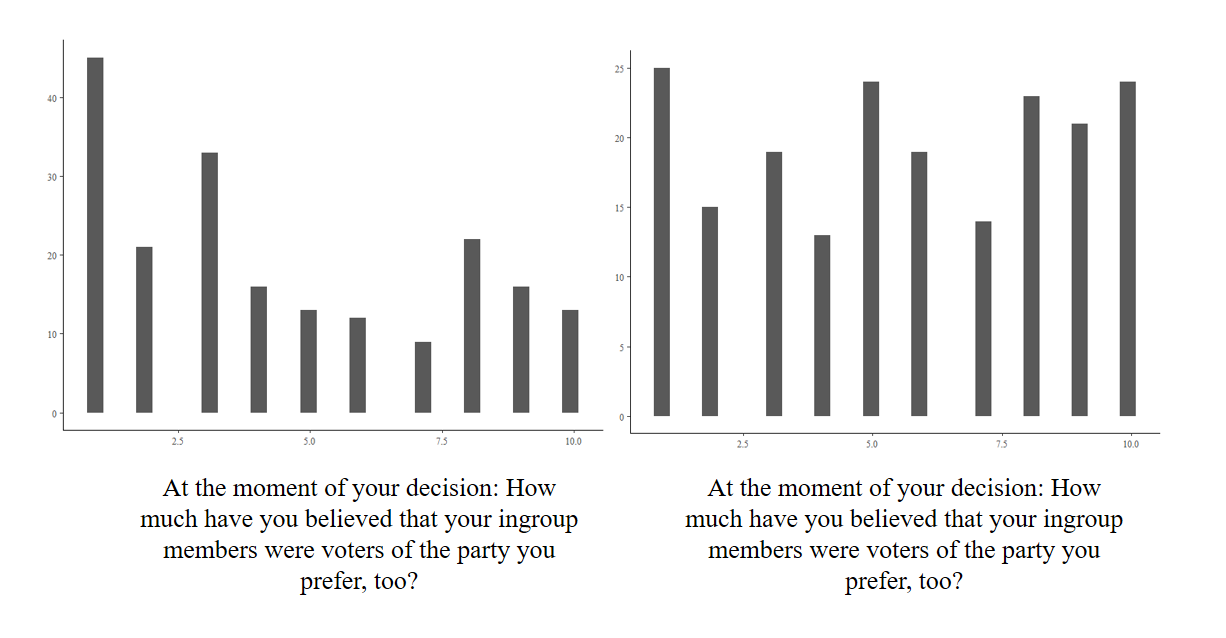


**Supplementary figure 2. Distribution of responses to belief item a (left histogram) and b (right histogram).**

**Supplementary table 3**

Results of robust Bayesian mixed linear model controlling for the effect of belief in identity of the ingroup and outgroup on decision patterns. Outgroup and ingroup beliefs critically predict within-group pool investments. The stress effect on IPD-MD investments was not moderated by belief.

| \|  \|  \|  \| \| \| \| --- \| --- \| --- \| --- \| --- \| \| Predictor \|  \| β \| 95%-lb \| 95%-ub \| \| Intercept \|  \| -0.06 \| -0.17 \| 0.04 \| \| Condition \|  \| 0.03 \| -0.15 \| 0.20 \| \| Pool \|  \| 0.79 \| 0.61 \| 0.97 \| \| Pool x Condition \|  \| -0.09 \| -0.44 \| 0.26 \| \| Ingroup Belief \|  \| -0.04 \| -0.14 \| 0.07 \| \| Outgroup Belief \|  \| 0.09 \| -0.01 \| 0.20 \| \| Ingroup Belief x Pool \|  \| -0.32 \| -0.53 \| -0.13 \| \| Outgroup Belief x Pool \|  \| 0.36 \| -0.16 \| -0.57 \| \| Ingroup Belief x Condition \|  \| -0.03 \| -0.23 \| 0.17 \| \| Outgroup Belief x Condition \|  \| 0.07 \| -0.15 \| 0.27 \| \| Ingroup Belief x Condition x Pool \|  \| 0.11 \| -0.30 \| 0.50 \| \| Outgroup Belief x Condition x Pool \|  \| 0.08 \| -0.32 \| 0.49 \| |  |  |
| --- | --- | --- | --- | --- | --- | --- | --- | --- | --- | --- | --- | --- | --- | --- | --- | --- | --- | --- | --- | --- | --- | --- | --- | --- | --- | --- | --- | --- | --- | --- | --- | --- | --- | --- | --- | --- | --- | --- | --- | --- | --- | --- | --- | --- | --- | --- | --- | --- | --- | --- | --- | --- | --- | --- | --- | --- | --- | --- | --- | --- | --- | --- | --- | --- | --- | --- | --- | --- | --- | --- | --- | --- |

*Trait Measures*

To ensure that there were no trait differences between the experimental groups in baseline and trait variables, we compared the trait variables between participants in the stress and the control group. As shown in supplementary table 5a (at the end of this document), participants in the stress and control groups only differed in chronotype (as measured with the MEQ) and the chronic stress subscale "Work Discontent" (as measured with the TICS) at an uncorrected alpha = .05 level. Given the number of comparisons, we consider these differences negligible. Nevertheless, we conducted moderation analyses to exclude potential effects of these a priori differences in chronotype and work discontent on our main analyses, but the results remain unchanged.

*Sex hormones*

We found no statistically significant differences in baseline sex hormone levels between the stress and control group, neither in testosterone (stress group: M = 46.93, *SD =* 40.8; control group: *M =* 45.13, *SD =* 43.61; *W* = 4,590.00, *p* = .739), estradiol (stress: *M =* 3.42, *SD =* 2.12; control: *M =* 3.64, *SD =* 1.85; *W* = 4,426.00, *p* = .161) nor in progesterone (stress: *M =* 25.61, *SD =* 45.40; control: *M =* 20.1, *SD =* 34.98; *W* = 4,829.50, *p* = .213). As expected, women had less saliva testosterone (males: *M =* 81.49, *SD =* 28.9; females: *M =* 8.6, *SD =* 5.64; W = 8,884.00, *p* < .001), but more progesterone than males (males: *M =* 6.22, *SD =* 7.26; females: *M =* 37.82, *SD =* 50.96; *W* = 1,475.50, *p* < .001). Salivary estradiol did not differ significantly between genders (males: *M =* 3.32, *SD =* 3.32; females: *M =* 3.74, *SD =* 2.09; *W* = 4,392.00, *p* = .138), but this conforms prior examinations of salivary sex hormone profiles^17,18^.

*Stress markers, sex hormones and investment patterns in the IPD-MD*

We conducted Bayesian parameter estimations to provide statistic ranges of sizes of the relevant effects, namely the main effect of condition (stress vs. control) and the interaction effect of condition x pool. In our main analyses, we chose non-informative priors (normally distributed [mean = 0, standard deviation = 10] priors for our fixed effects and t-distributions [three degrees of freedom, centered around zero and a scale parameter of 0.5] for the model intercept and the variance of the random intercept for each subject). Given our design and sample size, Bayesian parameter estimation is not very susceptible to change of priors at this sample size. To illustrate this, we present the results of the model with a range of different priors in supplementary table 4.

In the Bayesian hypothesis testing, we first estimated and compared models including the repeated measures factor pool, which, according to the significantly higher within-group pool investments compared to the between-group pool investments, should yield high Bayes factors in favor of the alternative hypotheses (BF10). Indeed, the models including pool alone, pool and stress condition, and the model incorporating the interaction term, yield BF10s over one-thousand. Adding the effect of pool to the null model reverses this pattern and yields the Bayes factors from the main analysis (only condition: BF01 = 8.523, “moderate” evidence^44^; condition and the interaction term condition x pool: BF01 = 51.476, “very strong” evidence^44^).

The choice of parameters in Bayesian Hypothesis Testing very much affects the outcomes. The r-scale specifies the alternative hypothesis with an r-scale of 1 assuming a huge effect –leading to high BF01 (for null hypothesis) if the effect is small or missing – and a small r-scale assuming a tiny effect, yielding increased BF10 (for the alternative hypothesis) if even a small difference is existent. To show that our analysis are not just a result of arbitrary choice (e.g. near-treshold) of r-scale, we calculated BF01s for different r-scale values (so called prior robustness tests, see figure supplementary figure 3). As it can be seen, evidence for the null-hypothesis occurs already for low r-scales (for both effects). Of course, this still does not prove that the effect is non-existent.

**Supplementary Table 4**

Results of Full Bayesian Model of Stress Effects on Pool Distributions depending on the choice of priors. All variables have been z-standardized.

|  |  |  |  |  |
| --- | --- | --- | --- | --- |
| Model | Prior for fixed effects | Parameter | Estimate | 95%-CrI |
|  |  |  |  |  |
| Ultra-Wide | normal(0, 10000) | condition (stress vs control) | 0.02 | [-0.17 : 0.19] |
| prior |  | pool x control | 0.08 | [-0.28 : 0.44] |
|  |  | pool (within & between groups) | -0.82 | [-1 : -0.65] |
| Wide prior | normal(0, 10) | condition (stress vs control) | 0.02 | [-0.17 : 0.19] |
|  |  | pool x control | 0.08 | [-0.28 : 0.44] |
|  |  | pool (within & between groups) | -0.82 | [-1 : -0.65] |
| Wide, but weakly | normal(0, 1) | condition (stress vs control) | 0.02 | [-0.17 : 0.21] |
| informative prior |  | pool x control | 0.07 | [-0.29 : 0.43] |
|  |  | pool (within & between groups) | -0.82 | [-0.99 : -0.64] |
| Informative | normal(0, 0.5) | condition (stress vs control) | 0.02 | [-0.16 : 0.20] |
| regularizing prior |  | pool x control | 0.07 | [-0.28 : 0.41] |
|  |  | pool (within & between groups) | -0.80 | [-0.97 : -0.62] |
| Tight informative | normal(0, 0.2) | condition (stress vs control) | 0.01 | [-0.15 : 0.18] |
| regularizing |  | pool x control | 0.04 | [-0.23 : 0.30] |
| prior |  | pool (within & between groups) | -0.68 | [-0.84 : -0.52] |
|  |  |  |  |  |

**Supplementary Figure 3. Bayes Factor robustness checks.** Both panels show an increase of Bayes factors for the null hypothesis (BF01) as a function of r scale for the fixed effects in the model – the scaling parameter that definies the shape of the model of the alternative hypothesis. **Panel a** illustrates the aforementioned increase for the main effect of stress, and **panel b** for the interaction effect between stress x pool. JASPs default r scale parameter is 0.5. Both panels show that evidence for the null hypothesis (> 3^19^ occurs already at a low r-scale, and thus, is not a product of arbitrary choice of priors.

Our parametric analyses reported in the main manuscript reveal that heart rate increase predicted a shift of investments from the within-group to the between-group pool, tentatively suggesting that heart rate increase as a proxy for sympathetic nervous system activation went along with higher outgroup-hate. To corroborate this conclusion, we accompanied our analyses with Student-t distribution-based robust Bayesian parameter estimations, which are less vulnerable to outliers. In a model containing the heart rate increase, pool, condition, as well as higher order interaction terms, the interaction between pool and heart rate increase points towards the same direction as the frequentist analysis in the main manuscript: The 95%-CrI posterior density of the interaction term estimate pool x heart rate increase ranges from 0.09 to .32, with a posterior estimate of .21. The other stress markers - CORT and α-amylase - do not predict allocations in the IPD-MD (pool x CORT interaction: β = -0.09, 95%-CrI = [-0.21 : 0.02]; and CORT alone: β = 0.06, 95%-CrI = [-0.06 : 0.17]; pool x α-amylase interaction: β = 0.02, 95%-CrI = [-0.09 : 0.14] and α-amylase alone: β = 0.04, 95%-CrI = [-0.16 : 0.09]). We used flat priors for the fixed effects. This analysis confirms that heart rate increase, but not stress or α-amylase, promoted a shift from within-group pool to between-group pool investments.

In further analyses, we report that, upon conditioning on testosterone, CORT significantly predicts within-group pool investments. Adding the same predictors – pool, condition, CORT, the respective sex hormone, the higher-order interactions and gender - to robust Bayesian models yields similar results: The 95%-CrI of our reported CORT x pool interaction is critically shifted away from 0 (β = -0.19, 95%-CrI = [-0.33 : -0.06]), and testosterone itself also critically predicts other-regarding preferences (β = 0.23, 95%-CrI = [0.04 : 0.42]). Unlike in our frequentist analyses, this model also indicates a critically shifted posterior for the β-estimate of CORT itself (β = 0.16, 95%-CrI = [0.02 : 0.30]). Also, a model including progesterone similarly yields a slightly shifted posterior interval for the CORT x pool interaction (β = -0.14, 95%-CrI = [-0.26 : -0.01]). Yet, testosterone and progesterone are correlated in male participants (rs = .265, p < .001), and it is not surprising that progesterone, up to some extent, adds similar information into the regression model.

**Further exploratory analyses**

*Hormonal and trait differences between genders*

A considerable amount of research finds sex hormones (mainly testosterone) to be strongly associated with shifts in altruism and competition^20–25^. In our IPD-MD, male participants displayed more in-group love and out-group hate than females, as reflected by higher within- and between-pool investments. One possible reason for this gender-difference in pool-investment might be the diverging sex hormone profiles across genders, but other reasons, such as gender-dependent differences in trait variables or societal or other factors are conceivable, too. We ran multiple mixed linear models for the between and within pool, condition, as well as gender and the sex hormones to test whether the baseline sex hormone compositions predicted pool investments. Neither testosterone, estradiol, progesterone, nor any of their linear interactions were associated with within-pool or between-pool investments (all p > .08). Mediation analyses provided no clear indication about a mediation of the gender effect via sex hormones. However, we would like to add that, due to high correlation between gender and sex-hormone levels, the power of such analyses might be decreased. Importantly, none of the sex hormones had predictive value when male and female participants were considered separately. Please notice that we did not enter CORT here as in the analysis mentioned in our manuscript, where testosterone eventually became a significant predictor.

Male and female participants differed in a number of trait variables: significant gender differences were found in in chronic stress (females > males), anxiety (tendency females > males), chronotype (males wake up later), risk taking (females < males), empathy (females > males), political interest and identification with the sympathized party (tendency females > males) as well as behavioral inhibition (females > males, see table supplementary table 5b at the end of this document). In a series of analyses, we tested whether these trait differences explained the gender effects on the pool-investments in the IPD-MD.

Because the gender effects only occurred in context of the abovementioned repeated measures ANOVA with pool and condition, we use a partialization approach to rule out potential influences of trait measures, in which we calculated residuals regressing each trait upon the between-group and within-group pool separately. In this way, we could partialize out the variance in our data explained by the trait variables while still maintaining the repeated measures structure of the IPD-MD design. Yet, the gender effects on pool investments mostly remained the same - male participants still invested more money into both pools than female participants (identification with party *F*(1, 197) = 5.88, *p* = .02, η_p_^2^= .03; psychopathy *F*(1, 197) = 5.88, *p* = .02, η_p_^2^ = .03; chronic stress *F*(1, 197) = 3.03, *p* = .08, η_p_^2^ = .02; chronotype *F*(1, 197) = 5.96, *p* = .02, η_p_^2^= .03, empathy *F*(1, 197) = 4.29, *p* = .04, η_p_^2^= .02), risk taking (*F*(1,197) = 5.07, *p* = .03, η_p_^2^= .025, behavioral inhibition *F*(1, 197) = 3.59, *p* = .06, η*_p_^2^* = .18, anxiety *F*(1, 197) = 3.90, *p* = .05, η*_p_^2^* = .015). We conclude that neither the gender differences in the sex hormone compositions, nor the differences in trait measures explained the gender effects on pool investments. Hence, these gender effects likely reflect factors not considered in this study, such as societal influences, social norms, gender stereotypes, group composition, or other variables.

*Traits associated with IPD-MD outcomes*

We further investigated the association of individual trait levels with investment patterns in the IPD-MD, and we tested whether some traits might have mediated, or obscured, the impact of stress on pool investments. To this end, we added the trait variables into a mixed linear model with condition (stress vs. control) x trait x pool (between vs. within) and a random intercept for each subject.

Besides chronic stress (see main text), we found high trait psychopathy to result in higher between-pool and lower within-pool contributions (pool x psychopathy interaction: β =0.201, *t*(394) = 4.693 , *p* < .001; main effect of psychopathy β = -0.118, *t*(394) = -2.747 , *p* = .006). Also, participants who were risk seeking invested more into the between-group pool and less into the within-group pool (pool x risk interaction: β =0.137, *t*(394) = 3.058 , *p* = .002). Behavioral activation, on the other hand, decreased within-group, but not between-group pool allocations (BAS x pool interaction β = .122, *t*(394) = 2.750, *p* = .006). More impulsive participants invested more into the within-group pool, and, hence, exhibited more ingroup-love (BAR x pool interaction β = -.0.096, *t*(394) = -2.291, *p* = .023). More ingroup love, but also less outgroup-hate was, unsurprisingly, also predicted by social value orientation (pool x social value orientation interaction β = -0.20, *t*(394) = -4.769 , *p* < .001, main effect of social value orientation β = 0.136, *t*(394) = 3.197, *p* = .002). However, none of the traits moderated the effect of stress on pool investments.

*IPD-MD allocations and cognitive reflection*

In addition to the IPD-MD, we also administered the Cognitive Reflection Test (CRT^46^) after the gTSST/control procedure, which has been shown to be CORT-sensitive^16^. To test whether stress-dependent cognitive reflection moderated (hidden) stress effects on pool investments, we ran additional analyses. Indeed, CRT performance was positively correlated with within-group pool investments (spearman correlation rs = .16, *p* = .027), but negatively correlated with between-group pool investments (spearman correlation rs = -.17, *p* = .018). This suggests that participants who made more reflective, less intuitive choices acted more in-group friendly. However, stress had no significant effect on CRT performance (*F*(1, 197) = 0, *p* = .97, η_p_^2^ < .001, suggesting that cognitive reflection modulated IPD-MD decisions independent of stress.

Hence, in sum, cognitive reflection was positively correlated with within- and negatively with between-group pool investments (cf. SOM for analyses). This result could be interpreted as evidence that the decisions in the IPD-MD game were dominated by deliberation over (social) emotion. Participants who were - by trait or state - in a cognitively deliberative mode after the stress procedure, invested more into the within-group pool.

**Supplementary Table 5a**

Comparison of traits between stress and control group. The default test was the Welch’s t-Test. Quantile-Quantile-Plots and histograms were used to diagnose violations of the normal distribution. In case of violation, we used the Wilcox rank sum test.

|  |  |  |  |  |  |  |  |
| --- | --- | --- | --- | --- | --- | --- | --- |
|  |  |  | Comparison between stress and control group | | | | |
|  |  |  |  |  |  |  |  |
| Measurement | Mean | SD | Condition | Mean | SD | Statistic | p-Value |
|  |  |  |  |  |  |  |  |
| BAR - Non-planning impulsiveness | 11.06 | 2.98 | Stress | 10.79 | 2.89 |  |  |
|  |  |  | Control | 11.32 | 3.05 | -1.28 | 0.203 |
| BAR - Motoric Impulsiveness | 10.68 | 2.45 | Stress | 10.55 | 2.36 |  |  |
|  |  |  | Control | 10.81 | 2.54 | -0.78 | 0.438 |
| BAR - Attention Impulsiveness | 9.44 | 2.4 | Stress | 9.38 | 2.61 |  |  |
|  |  |  | Control | 9.5 | 2.2 | -0.34 | 0.734 |
| BAR Total | 31.18 | 5.79 | Stress | 30.72 | 5.59 |  |  |
|  |  |  | Control | 31.64 | 5.97 | -1.13 | 0.261 |
| BAS - Drive | 12.46 | 1.91 | Stress | 12.46 | 1.81 |  |  |
|  |  |  | Control | 12.45 | 2.02 | 0.05 | 0.96 |
| BAS - Funseeking | 12.33 | 1.77 | Stress | 12.39 | 1.87 |  |  |
|  |  |  | Control | 12.27 | 1.67 | W = 5278.5 | 0.573 |
| BAS - Reward responsiveness | 16.78 | 1.89 | Stress | 16.93 | 2.05 |  |  |
|  |  |  | Control | 16.63 | 1.73 | W = 5601.5 | 0.176 |
| BIS Total | 19.87 | 3.78 | Stress | 19.51 | 3.73 |  |  |
|  |  |  | Control | 20.22 | 3.81 | -1.33 | 0.184 |
| BAS Total | 41.57 | 4.27 | Stress | 41.79 | 4.56 |  |  |
|  |  |  | Control | 41.35 | 3.98 | W = 5311 | 0.524 |
| SDS Total | 21.92 | 3.68 | Stress | 21.96 | 2.8 |  |  |
|  |  |  | Control | 21.88 | 2.58 | 0.2 | 0.839 |
| TICS Work Overload | 21.51 | 5.96 | Stress | 21.71 | 5.89 |  |  |
|  |  |  | Control | 21.32 | 6.05 | 0.46 | 0.649 |
| TICS Social Overload | 14.33 | 4.54 | Stress | 14.78 | 4.42 |  |  |
|  |  |  | Control | 13.89 | 4.63 | 1.39 | 0.167 |
| TICS Pressure to perform | 25.65 | 5.33 | Stress | 25.92 | 5.61 |  |  |
|  |  |  | Control | 25.38 | 5.07 | 0.71 | 0.478 |
| TICS Work Discontent | 21.34 | 5.42 | Stress | 20.46 | 5.48 |  |  |
|  |  |  | Control | 22.19 | 5.24 | -2.27 | 0.024 |
| TICS Excessive Demands at Work | 13.22 | 4.13 | Stress | 13.16 | 4.23 |  |  |
|  |  |  | Control | 13.28 | 4.05 | W = 4930 | 0.773 |
| TICS Lack of Social Recognition | 9.99 | 2.79 | Stress | 9.9 | 2.73 |  |  |
|  |  |  | Control | 10.08 | 2.87 | -0.45 | 0.65 |
| TICS Social Tensions | 13.32 | 4.41 | Stress | 13.12 | 4.4 |  |  |
|  |  |  | Control | 13.51 | 4.43 | W = 4804 | 0.552 |
| TICS Social Isolation | 14.6 | 4.95 | Stress | 14.2 | 5.03 |  |  |
|  |  |  | Control | 14.98 | 4.87 | W = 4626.5 | 0.305 |
| TICS Chronic Worrying | 11 | 3.49 | Stress | 10.85 | 3.59 |  |  |
|  |  |  | Control | 11.14 | 3.41 | -0.58 | 0.56 |
| TICS Total | 144.77 | 27.25 | Stress | 143.86 | 28.11 |  |  |
|  |  |  | Control | 145.66 | 26.5 | -0.47 | 0.641 |
| STAI Total | 51.96 | 7.4 | Stress | 51.63 | 8.22 |  |  |
|  |  |  | Control | 52.27 | 6.54 | -0.62 | 0.538 |
| LSRP Psychopathic Affect | 26.33 | 6.17 | Stress | 26.4 | 6.56 |  |  |
|  |  |  | Control | 26.25 | 5.79 | W = 5049.5 | 1 |
| LSRP Psychopathic Lifestyle | 20.05 | 3.4 | Stress | 19.76 | 3.28 |  |  |
|  |  |  | Control | 20.34 | 3.5 | -1.22 | 0.222 |
| LSRP Total | 49.51 | 8.3 | Stress | 49.31 | 8.54 |  |  |
|  |  |  | Control | 49.71 | 8.1 | W = 4917.5 | 0.750 |
| Chronotype | 12.4 | 3.71 | Stress | 13.03 | 3.72 |  |  |
|  |  |  | Control | 11.79 | 3.61 | 2.39 | 0.018 |
| SVO_prosocial | 6.21 | 3.76 | Stress | 6.09 | 3.83 |  |  |
|  |  |  | Control | 6.32 | 3.69 | W = 4836 | 0.5677 |
| Party Affiliation | 2.51 | 0.69 | Stress | 2.45 | 0.69 |  |  |
|  |  |  | Control | 2.57 | 0.7 | W = 4604.5 | 0.23 |
| Political identification | 2.75 | 0.64 | Stress | 2.78 | 0.63 |  |  |
|  |  |  | Control | 2.71 | 0.65 | 0.81 | 0.417 |
| SPF Fantasy | 13.12 | 3.31 | Stress | 13.14 | 3.43 |  |  |
|  |  |  | Control | 13.11 | 3.2 | 0.07 | 0.943 |
| SPF Empathetic Concern | 14.22 | 2.6 | Stress | 14.27 | 2.77 |  |  |
|  |  |  | Control | 14.18 | 2.44 | W = 5500 | 0.269 |
| SPF Perspective Takng | 14.84 | 2.67 | Stress | 14.95 | 2.54 |  |  |
|  |  |  | Control | 14.74 | 2.8 | 0.57 | 0.57 |
| SPF Personal Distress | 10.29 | 2.77 | Stress | 10.16 | 2.77 |  |  |
|  |  |  | Control | 10.41 | 2.77 | -0.64 | 0.523 |
| SPF Empathy Score | 42.19 | 6.46 | Stress | 42.36 | 6.74 |  |  |
|  |  |  | Control | 42.02 | 6.21 | W = 5411 | 0.3798 |
| Risk Taking | 1.81 | 0.91 | Stress | 1.89 | 0.99 |  |  |
|  |  |  | Control | 1.72 | 0.81 | W = 5450 | 0.2939 |
|  |  |  |  |  |  |  |  |

**Supplementary Table 5b**

Comparison between male and female participants in trait measures. The default test was the Welch’s t-Test. Quantile-Quantile-Plots and histograms were used to diagnose violations of the normal distribution. In case of violation, we used the Wilcox rank sum test.

|  |  |  |  |  |  |  |  |
| --- | --- | --- | --- | --- | --- | --- | --- |
|  |  |  | Comparson between genders | | | | |
|  |  |  |  |  |  |  |  |
| Measurement | Mean | SD | Gender | Mean | SD | Statistic | p-Value |
|  |  |  |  |  |  |  |  |
| BAR - Non-planning impulsiveness | 11.06 | 2.98 | Male | 11.3 | 2.99 |  |  |
|  |  |  | Female | 10.82 | 2.97 | 1.14 | 0.257 |
| BAR - Motoric Impulsiveness | 10.68 | 2.45 | Male | 10.59 | 2.4 |  |  |
|  |  |  | Female | 10.77 | 2.51 | -0.53 | 0.599 |
| BAR - Attention Impulsiveness | 9.44 | 2.4 | Male | 9.7 | 2.6 |  |  |
|  |  |  | Female | 9.19 | 2.18 | 1.51 | 0.132 |
| BAR Total | 31.18 | 5.79 | Male | 31.59 | 6.08 |  |  |
|  |  |  | Female | 30.78 | 5.48 | 0.99 | 0.324 |
| BAS - Drive | 12.46 | 1.91 | Male | 12.2 | 2.07 |  |  |
|  |  |  | Female | 12.71 | 1.72 | -1.91 | 0.057 |
| BAS - Funseeking | 12.33 | 1.77 | Male | 12.39 | 1.75 |  |  |
|  |  |  | Female | 12.28 | 1.8 | W = 5275 | 0.581 |
| BAS - Reward responsiveness | 16.78 | 1.89 | Male | 16.64 | 1.93 |  |  |
|  |  |  | Female | 16.91 | 1.86 | W = 4718 | 0.416 |
| BIS Total | 19.87 | 3.78 | Male | 18.59 | 3.64 |  |  |
|  |  |  | Female | 21.13 | 3.5 | -5.04 | 0.001 |
| BAS Total | 41.57 | 4.27 | Male | 41.23 | 4.45 |  |  |
|  |  |  | Female | 41.9 | 4.08 | W = 4622.5 | 0.299 |
| SDS Total | 21.92 | 3.68 | Male | 22.02 | 2.79 |  |  |
|  |  |  | Female | 21.82 | 2.59 | 0.52 | 0.602 |
| TICS Work Overload | 21.51 | 5.96 | Male | 19.62 | 5.23 |  |  |
|  |  |  | Female | 23.39 | 6.06 | -4.72 | 0.001 |
| TICS Social Overload | 14.33 | 4.54 | Male | 13.44 | 4.18 |  |  |
|  |  |  | Female | 15.21 | 4.73 | -2.81 | 0.005 |
| TICS Pressure to perform | 25.65 | 5.33 | Male | 25.35 | 5.52 |  |  |
|  |  |  | Female | 25.94 | 5.16 | -0.78 | 0.434 |
| TICS Work Discontent | 21.34 | 5.42 | Male | 21.59 | 6.17 |  |  |
|  |  |  | Female | 21.09 | 4.57 | 0.65 | 0.514 |
| TICS Excessive Demands at Work | 13.22 | 4.13 | Male | 12.4 | 4.15 |  |  |
|  |  |  | Female | 14.04 | 3.95 | W = 3797 | <0.001 |
| TICS Lack of Social Recognition | 9.99 | 2.79 | Male | 9.61 | 2.85 |  |  |
|  |  |  | Female | 10.37 | 2.7 | -1.93 | 0.055 |
| TICS Social Tensions | 13.32 | 4.41 | Male | 13.37 | 4.4 |  |  |
|  |  |  | Female | 13.27 | 4.44 | W = 5125 | 0.8562 |
| TICS Social Isolation | 14.6 | 4.95 | Male | 14.44 | 5.05 |  |  |
|  |  |  | Female | 14.75 | 4.87 | W = 4808 | 0.557 |
| TICS Chronic Worrying | 11 | 3.49 | Male | 10.3 | 3.36 |  |  |
|  |  |  | Female | 11.68 | 3.51 | -2.86 | 0.005 |
| TICS Total | 144.77 | 27.25 | Male | 139.81 | 27.43 |  |  |
|  |  |  | Female | 149.68 | 26.29 | -2.6 | 0.01 |
| STAI Total | 51.96 | 7.4 | Male | 50.93 | 7.46 |  |  |
|  |  |  | Female | 52.97 | 7.25 | -1.97 | 0.051 |
| LSRP Psychopathic Affect | 26.33 | 6.17 | Male | 27.21 | 6.51 |  |  |
|  |  |  | Female | 25.46 | 5.71 | W = 5745.5 | 0.091 |
| LSRP Psychopathic Lifestyle | 20.05 | 3.4 | Male | 20 | 3.33 |  |  |
|  |  |  | Female | 20.11 | 3.48 | -0.23 | 0.821 |
| LSRP Total | 49.51 | 8.3 | Male | 50.59 | 8.4 |  |  |
|  |  |  | Female | 48.45 | 8.11 | W = 5686 | 0.122 |
| Chronotype | 12.4 | 3.71 | Male | 11.59 | 3.72 |  |  |
|  |  |  | Female | 13.21 | 3.53 | -3.16 | 0.002 |
| SVO prosocial | 6.21 | 3.76 | Male | 6.28 | 3.75 |  |  |
|  |  |  | Female | 6.14 | 3.77 | W = 5070 | 0.95 |
| Party Affiliation | 2.51 | 0.69 | Male | 2.4 | 0.64 |  |  |
|  |  |  | Female | 2.62 | 0.73 | W = 4400.5 | 0.077 |
| Political identification | 2.75 | 0.64 | Male | 2.79 | 0.63 |  |  |
|  |  |  | Female | 2.7 | 0.65 | 1.02 | 0.311 |
| SPF Fantasy | 13.12 | 3.31 | Male | 12.33 | 3.22 |  |  |
|  |  |  | Female | 13.91 | 3.22 | -3.48 | 0.001 |
| SPF Empathetic Concern | 14.22 | 2.6 | Male | 13.45 | 2.72 |  |  |
|  |  |  | Female | 14.99 | 2.24 | W = 3325 | < 0.001 |
| SPF Perspective Takng | 14.84 | 2.67 | Male | 14.63 | 2.71 |  |  |
|  |  |  | Female | 15.05 | 2.62 | -1.11 | 0.267 |
| SPF Personal Distress | 10.29 | 2.77 | Male | 9.17 | 2.47 |  |  |
|  |  |  | Female | 11.4 | 2.6 | -6.22 | 0.001 |
| SPF Empathy Score | 42.19 | 6.46 | Male | 40.41 | 6.47 |  |  |
|  |  |  | Female | 43.95 | 5.98 | W = 3473 | < 0.001 |
| Risk Taking | 1.81 | 0.91 | Male | 1.97 | 0.97 |  |  |
|  |  |  | Female | 1.63 | 0.81 | W = 6071 | 0.007 |
|  |  |  |  |  |  |  |  |

**References (appendix only)**

1. Juster, R.-P., Vencill, J. A. & Johnson, P. J. Impact of Stress and Strain on Current LGBT Health Disparities. in *Trauma, Resilience, and Health Promotion in LGBT Patients: What Every Healthcare Provider Should Know* (eds. Eckstrand, K. L. & Potter, J.) 35–48 (Springer International Publishing, 2017). doi:10.1007/978-3-319-54509-7_4

2. Kirschbaum, C., Pirke, K.-M. & Hellhammer, D. H. The ‘Trier Social Stress Test’ – A Tool for Investigating Psychobiological Stress Responses in a Laboratory Setting. *NPS* **28**, 76–81 (1993).

3. Von Dawans, B., Ditzen, B., Trueg, A., Fischbacher, U. & Heinrichs, M. Effects of acute stress on social behavior in women. *Psychoneuroendocrinology* **99**, 137–144 (2019).

4. Ohr, D. Parteiidentifikation in Deutschland: Eine empirische Fundierung des Konzepts auf Basis der Theorie Sozialer Identität. in *Wählen in Deutschland* 186–209 (Nomos Verlagsgesellschaft mbH & Co. KG, 2011).

5. Meule, A., Vögele, C. & Kübler, A. Psychometrische Evaluation der deutschen Barratt Impulsiveness Scale – Kurzversion (BIS-15). *Diagnostica* **57**, 126–133 (2011).

6. Carver, C. S. & White, T. L. Behavioral inhibition, behavioral activation, and affective responses to impending reward and punishment: The BIS/BAS Scales. *Journal of Personality and Social Psychology* **67**, 319–333 (1994).

7. Stöber, J. The Social Desirability Scale-17 (SDS-17). *European Journal of Psychological Assessment* **17**, 222–232 (2001).

8. Spielberger, C. D. Manual for the State-Trait Anxiety Inventory STAI (Form Y) (‘Self-Evaluation Questionnaire’). (1983).

9. Van Lange, P. A. M., De Bruin, E., Otten, W. & Joireman, J. A. Development of prosocial, individualistic, and competitive orientations: Theory and preliminary evidence. **74**, 733–746 (1997).

10. Levenson, M. R., Kiehl, K. A. & Fitzpatrick, C. M. Assessing psychopathic attributes in a noninstitutionalized population. *J Pers Soc Psychol* **68**, 151–158 (1995).

11. Davis, M. H. The effects of dispositional empathy on emotional reactions and helping: A multidimensional approach. *Journal of personality* **51**, 167–184 (1983).

12. Paulus, C. Der Saarbrücker Persönlichkeitsfragebogen SPF(IRI) Zur Messung von Empathie: Psychometrische Evaluation der deutschen Version des Interpersonal Reactivity Index. (2009).

13. Randler, C. German version of the reduced Morningness–Eveningness Questionnaire (rMEQ). *Biological rhythm research* **44**, 730–736 (2013).

14. Schulz, P. & Schlotz, W. The Trier Inventory for the Assessment of Chronic Stress (TICS). Scale construction, statistical testing, and validation of the scale work overload. *Diagnostica* **45**, 8–19 (1999).

15. Frederick, S. Cognitive Reflection and Decision Making. *Journal of Economic Perspectives* **19**, 25–42 (2005).

16. Margittai, Z. *et al.* Exogenous cortisol causes a shift from deliberative to intuitive thinking. *Psychoneuroendocrinology* **64**, 131–135 (2016).

17. Shirtcliff, E. A., Granger, D. A. & Likos, A. Gender differences in the validity of testosterone measured in saliva by immunoassay. *Hormones and Behavior* **42**, 62–69 (2002).

18. Gavrilova, N. & Lindau, S. T. Salivary Sex Hormone Measurement in a National, Population-Based Study of Older Adults. *J Gerontol B Psychol Sci Soc Sci* **64B**, i94–i105 (2009).

19. Kass, R. E. & Raftery, A. E. Bayes Factors. *Journal of the American Statistical Association* **90**, 773–795 (1995).

20. Carré, J. M., Putnam, S. K. & McCormick, C. M. Testosterone responses to competition predict future aggressive behaviour at a cost to reward in men. *Psychoneuroendocrinology* **34**, 561–570 (2009).

21. Anderl, C. *et al.* Cooperative preferences fluctuate across the menstrual cycle. *Judgment and Decision Making* **10**, 400- (2015).

22. Losecaat Vermeer, A. B., Riečanský, I. & Eisenegger, C. Competition, testosterone, and adult neurobehavioral plasticity. in *Progress in Brain Research* (eds. Studer, B. & Knecht, S.) 213–238 (Elsevier, 2016).

23. Geniole, S. N., Bird, B. M., Ruddick, E. L. & Carré, J. M. Effects of competition outcome on testosterone concentrations in humans: An updated meta-analysis. *Hormones and Behavior* **92**, 37–50 (2017).

24. Dreher, J.-C. *et al.* Testosterone causes both prosocial and antisocial status-enhancing behaviors in human males. *PNAS* **113**, 11633–11638 (2016).

25. Mehta, P. H., Lawless DesJardins, N. M., van Vugt, M. & Josephs, R. A. Hormonal underpinnings of status conflict: Testosterone and cortisol are related to decisions and satisfaction in the hawk-dove game. *Hormones and Behavior* **92**, 141–154 (2017).
